# Conformational studies of biaryl-bridged seven-membered lactones with a quaternary carbon center†

**DOI:** 10.1039/d4ra04703f

**Published:** 2024-08-14

**Authors:** Yu Zhang, Yueyang Yu, Xiaoqiang Ma, Xiaofei Zhao, Jialin Huang, Depeng Zhao

**Affiliations:** a State Key Laboratory of Anti-infective Drug Discovery and Development, School of Pharmaceutical Sciences, Sun Yat-sen University Guangzhou 510006 China zhaodp5@mail.sysu.edu.cn

## Abstract

We show the synthesis and conformational studies of a series of 7,7-disubstituted-dibenzo[*b*,*d*]oxepin-6(7*H*)-ones that feature biaryl-bridged seven-membered lactones with a quaternary carbon center, in which the larger substituents prefer the axial positions. Further studies on the crystal structures and DFT calculations revealed that the high selectivity observed is attributed to the volume of substituents.

## Abstract 2

Here, conformations of a series of 7,7-disubstituted-dibenzo[*b*,*d*]oxepin-6(7*H*)-ones were studied and we found that the stable conformers all favor to place the larger group in the axial position instead of the equatorial position.

## Graphical abstract

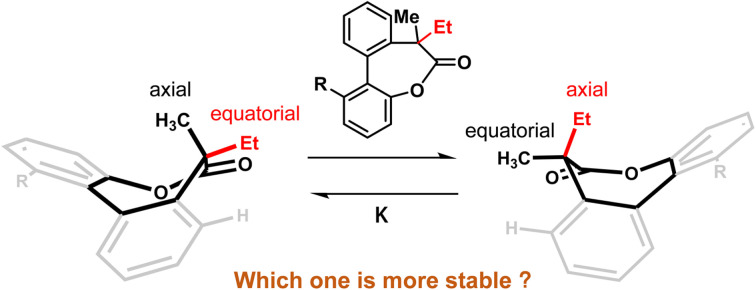

## Introduction

**Fig. 1:**
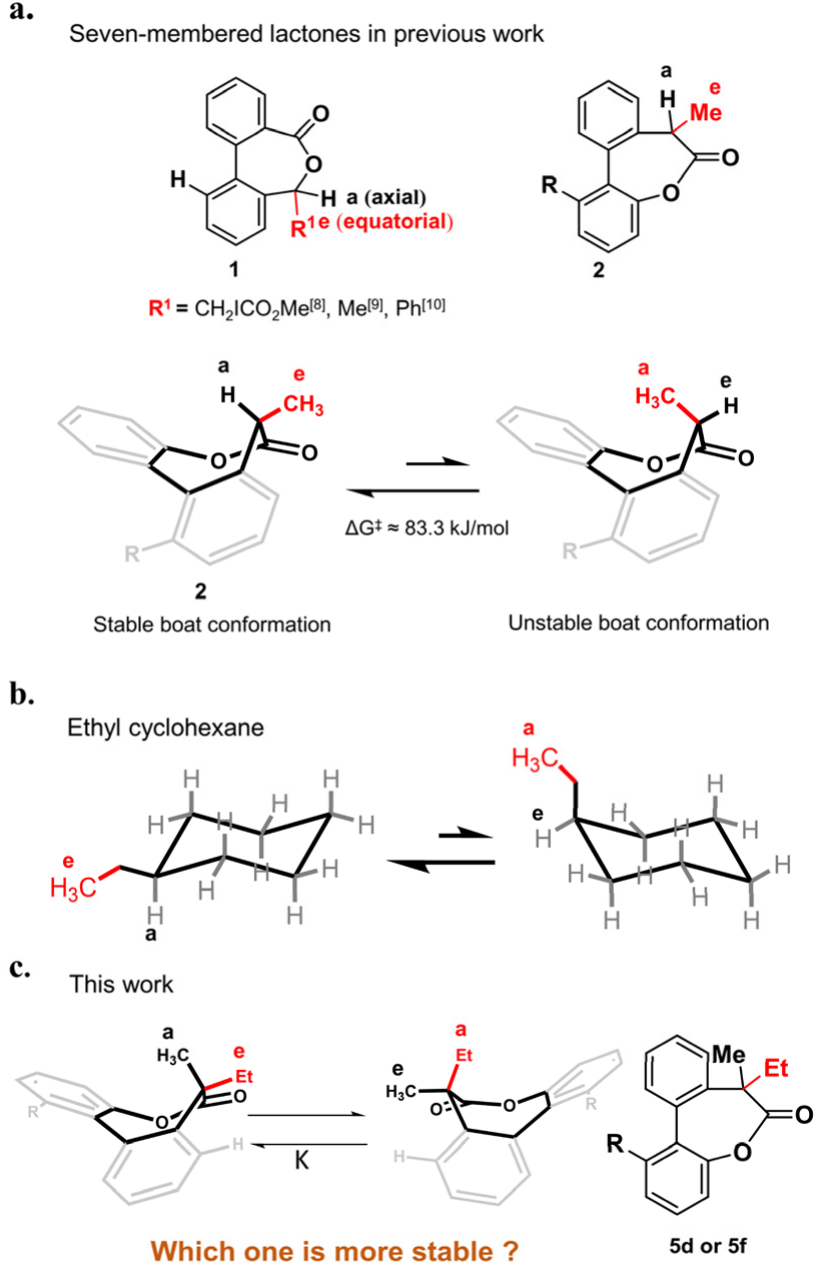
(a) Conformations of seven-membered lactones with tertiary carbon in previous work. (b) Two chair conformations of ethyl cyclohexane. (c) Two conformations of the biaryl-bridged seven-membered lactone 5d or 5f with a quaternary carbon center.

Substituted hexane is the earliest model investigated for conformational analysis of cyclic compounds.^1–4^ Take the ethyl cyclohexane as an example (Fig. 1b), it has two chair conformations in which the conformation with the ethyl in the equatorial position is more stable as a result of the steric strain in the axial position.^5^ The ratio of the two conformations is determined by the energy difference of them, which is defined as the *A* value in the cyclohexane system.^6,7^ The *A* value principle is not limited to substituted hexanes but also applies to other ring systems.^8–11^ For example, a few seven-membered chiral lactones 1 derived from chiral alcohols with low barriers of inversion have been reported^8–10^ in the last few years and the stable conformations also favor to place the larger *R* group in the equatorial position as confirmed by the crystal structures (Fig. 1a). As seven-membered ring lactones have shown some effective biological activities such as antitumor effects^12–14^ and can be used as a dynamic covalent bond to build dynamic molecular system, our group has synthesized a chiral lactone 2 derived from chiral carboxylic acid to build molecular motors.^11^ The stable conformation of compound 2 was identified by single crystal analysis as well, in which the larger methyl group was also in the equatorial position while the smaller hydrogen was in the axial position (Fig. 1a). To date, most of the conformational studies of cyclic compounds are limited to tertiary carbon^15–17^ and cyclic systems with a quaternary carbon center have been less studied.^18^ Only a few 1,1-disubstituted cyclohexanes^19–21^ have been studied and the *A* value principle is still applicable to them. The conformations where the larger substituents adopt the equatorial positions are more stable except for the presence of phenyl groups.^21–24^ As an exception, 1-methyl-1-phenylcyclohexane or 1-isopropyl-1-phenylcyclohexane preferentially adopts the conformation with an axial phenyl group as the rotation of the equatorial phenyl group in the symmetry planes of the cyclohexane rings is perturbed by the geminal methyl or isopropyl group.^20,21^

Following our longstanding interest in the helical chirality of cyclic biphenyls induced by the chiral center in lactone structures, we questioned if the hydrogen of the tertiary carbon in 2 was replaced by substituents larger than the methyl group to afford a quaternary carbon, what about the stereoselectivity and which one is the more stable conformation (Fig. 1c). Herein, we present our preliminary study on the synthesis and conformational studies of a series of the 7,7-disubstituted-dibenzo[*b*,*d*]oxepin-6(7*H*)-ones. The results clearly indicate that the volume of substituents dominate the high preference of the axial position for the larger substituents over the methyl group in this system.

## Results and discussion

**Scheme 1:**
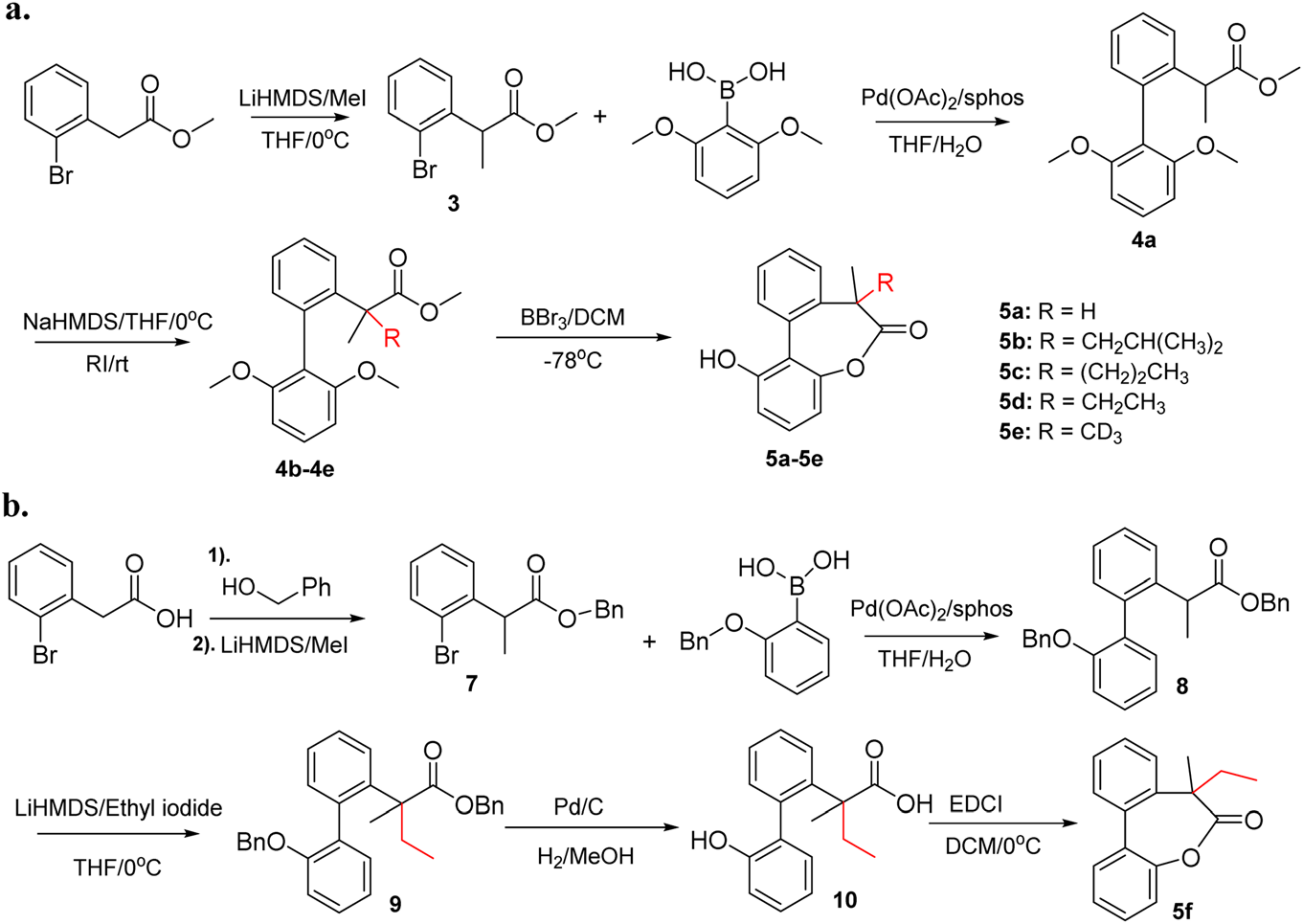
(a) Synthetic procedures for 5a–5e with biaryl-bridged lactone structures. (b) Synthetic procedures for 5f.

The experimental procedures for chemical synthesis of compound 5 are given in Scheme 1. Compound 5a was reported in our previous work and shows high preference (>20 : 1) of one helicity due to the chiral induction of the tertiary carbon.^11^ The rest of the compounds were synthesized using an identical approach as shown in Scheme 1a. Compound 3 was obtained by methylation of methyl 2-(2-bromophenyl)acetate with lithium bis(trimethylsilyl)amide and iodomethane. Suzuki coupling of 3 and (2,6-dimethoxyphenyl)boronic acid afforded a common intermediate 4a. Subsequently, 4a was treated with sodium bis(trimethylsilyl)amide and the corresponding alkylating reagent to obtain 4b–4e with a quaternary center. Finally, lactones 5b–5e were obtained by demethylation and cyclization of 4b–4e with BBr_3_. More details about the synthetic procedures are given in the ESI.†

To study the effect of hydroxyl group of the biphenyls on the stereoselectivity, 5f without the free hydroxyl group was also synthesized. As shown in Scheme 1b, benzyl alcohol was used to protect the carboxylic acid to facilitate deprotection of benzyl ether and benzyl ester in one step. Using an identical approach, methylation and Suzuki coupling afforded 8 which underwent a second alkylation with ethyl iodide to get biphenyl 9. The targeted lactone 5f was synthesized by deprotection of benzyl groups followed by cyclization with EDCI (1-Ethyl-3-(3-dimethylaminopropyl) carbodiimide).

**Fig. 2:**
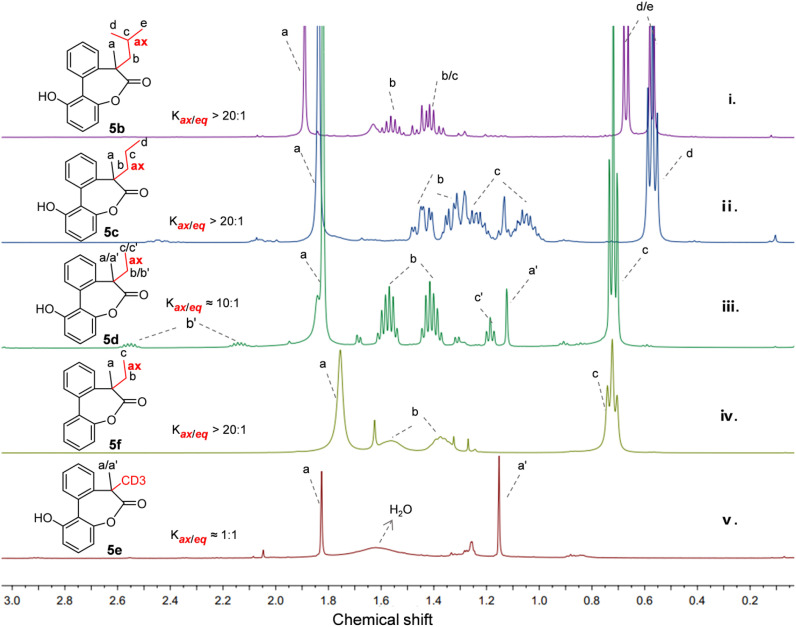
Partial ^1^H NMR spectra of the compounds 5b–5f in CDCl_3_ at 298 K.

**Fig. 3:**
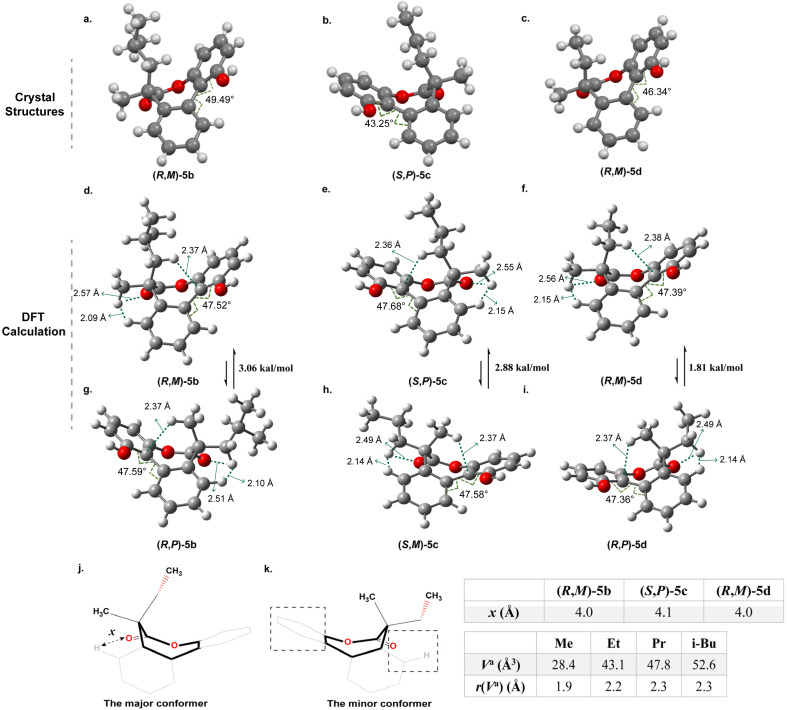
The crystal structures and optimized structures by DFT calculation of 5b–5d. (a to c) The single crystal structure of 5b to 5d; (d to f) The optimized structures of the 5b to 5d with the larger groups in the axial positions; (g to i) the optimized structures of the 5b–5d with the larger groups in the equatorial positions; (j and k) the two boat conformation of the 5d (*x* denotes the distance between the carbonyl oxygen atom and hydrogen atom in the benzene ring measured from the crystal structures).

Taking the size of the substituents into account, lactone 5b with an isobutyl group was firstly studied. As shown in Fig. 2(i), in the presence of the methyl and isobutyl on the quaternary carbon of the biaryl-bridged seven-membered lactones, high selectivity between two conformers with axial or equatorial isobutyl (*K*_ax/eq_ > 20 : 1) was observed and almost only one set of peaks appeared in the ^1^H NMR spectrum. Fortunately, a single crystal of 5b was obtained and the crystal structure is given in Fig. 3a. Remarkably, we noticed that the methyl adopted an equatorial position in the dominant conformation of 5b while the larger isobutyl group adopted an axial position, which is distinct from the case of 5a or 2 with a tertiary carbon reported previously in which the smaller hydrogen was in the axial position while the larger methyl group was in the equatorial bond.^11^ In the case of 5c with a methyl and a *n*-propyl, a ratio greater than 20 : 1 was also observed from the ^1^H NMR spectrum (Fig. 2(ii)). Likewise, the single crystal structure of 5c (Fig. 3b) revealed a stable conformation identical to 5b, with the larger *n*-propyl group occupying the axial position.

Under the consideration of that the ethyl has similar *A* value of 7.49 kJ mol^−1^ with methyl of 7.28 kJ mol^−1^,^7^ we then reduced the size of the substituent again and studied 5d using ^1^H NMR and X-ray diffraction analysis. Surprisingly, a high 10 : 1 ratio of conformers with axial ethyl and equatorial ethyl was achieved as shown in Fig. 2(iii). The ethyl group on the molecule was also confirmed to occupy the axial position by single crystal structure (Fig. 3c). This cyclic system appears to be highly sensitive to the size of the substituents. To rule out the possibility that lactones 5b–5f are kinetically trapped isomers, we calculated the energy barrier for flipping from the unstable conformers to stable ones in chloroform using density functional theory (DFT) at the SMD(chloroform)-ωB97X-D/def2-TZVP level of theory. The resulting activation energy barriers of 5b–5d are 71.3, 73.4 and 74.0 kJ mol^−1^ at 298 K, respectively, which are consistent with the results for 2 in our previous work and other related structures.^25,26^ The low activation energy barriers obtained confirm that the ratios observed in the ^1^H NMR spectra of 5b–5d in Fig. 2 represent the equilibrium ratio. For the lactone 5f, almost only one set of peaks can be seen in the ^1^H NMR spectrum at room temperature (Fig. 2(iv)). Furthermore, the low-temperature NMR experiments to slow down the interconversion of conformers were carried out due to the appearance of broad lines. However, the ^1^H NMR spectra was obtained and still only one set of peaks can be seen at −50 °C in deuterated chloroform and −90 °C in deuterated methanol, as shown in Fig. S1 and S2.† Also, the calculated activation energy barrier for 5f is 51.5 kJ mol^−1^ at 298 K.

The single-crystal structures of 5b–5d reveal that the seven-membered lactones adopt a boat conformation with the larger group in the axial position (Fig. 3a–c). This phenomenon is not common and distinct from the situation in 5a with a tertiary carbon and previous examples.^8,10,11^ The twist angles *θ* in structures 5b, 5c and 5d are 49.5°, 43.3° and 46.3°, respectively. Details for crystallographic data are provided in Tables S1–S3.†

Following the study of progressively smaller substituents and driven by the curiosity about the minimal difference between hydrogen and deuterium,^27,28^ we next investigated compound 5e, which features a methyl and a deuterated methyl group on the quaternary carbon. The ^1^H NMR spectrum of 5e obtained in CDCl_3_ shows a ratio of 1 : 1 of two conformers approximately and the chemical shift of the protons of the methyl group in two conformers are 1.82 ppm and 1.15 ppm respectively (Fig. 2(v)). As for 5a where the methyl group was in equatorial bond, the chemical shift of the methyl was about 1.67 ppm.^11^ However, in the case of 5e, when the methyl group is in the axial position pointing toward the shielding zone of the phenyl ring, the chemical shift of the methyl group shifts upfield with emergence of the signal of the protons at *δ* 1.15 ppm due to the corresponding shielding effects.^29–31^ A similar upfield-shift was also observed for 5d. The chemical shift of the methyl group of the major conformer with the equatorial methyl group is 1.82 ppm and the minor conformer with the methyl group in the axial position shows an upfield shift to 1.12 ppm (Fig. 2(iii)).

To further understand the above experimental results, DFT calculations were performed using the Gaussian16, Revision A.03 at the SMD(chloroform)-ωB97X-D/def2-TZVP level of theory, which aimed to identify the stable conformer in the system and make sense of the correlation between the size of the substituents and the stereoselectivity. As for the optimized structures of the (*R*,*M*)-5b and the (*R*,*P*)-5b, the calculated results indicated that the (*R*,*M*)-5b with an axial isobutyl is thermodynamically more stable than (*R*,*P*)-5b with the equatorial isobutyl (Fig. 3d and g). The calculated energy difference between the two conformers is 3.06 kcal mol^−1^ approximately, it can be sure of the position of the isobutyl to be axial. The theoretical equilibrium proportion for 5b is close to 174 : 1 through the calculation by the Gibbs free energy formula (Δ*G*° = −*RT* ln *K*_eq_), which indicates that the conformer with axial isobutyl is almost exclusively favoured over the one with equatorial isobutyl. It is in agreement with the experimental result of only one set of peaks obtained in the ^1^H NMR spectrum (Fig. 2(i)). And it is the same cas for 5c and 5d (Fig. 3e, h, f and i), which also show obvious preference for placing the larger substituents in the axial positions rather than the equatorial positions. Furthermore, the energy difference between the stable conformer and unstable conformer of 5c and 5d is 2.88 and 1.81 kal mol^−1^ respectively in calculations (Fig. 3). This observation is consistent with the single crystal structures illustrated in Fig. 3b and c and the results of ^1^H NMR experiments (Fig. 2(ii) and (iii)). It is obvious that the energy difference decreases gradually with the decrease of size of the substituents, which corresponds to the trend of stereoselectivity. In other words, it is apparent that as the steric hindrance of the substituent increases, there is a corresponding increase in the energy difference and stereoselectivity between the two different conformations with axial or equatorial larger substituent on the seven-membered rings.

However, we questioned why the larger groups were on the axial positions in such biaryl-bridged seven-membered lactones with quaternary carbons, which is distinct from previous studies with tertiary carbons. Take 5d as an example, two boat conformations from the simulated structures are shown in Fig. 3j and k. It is consistent with the single crystal structures of previous studies with tertiary carbons, where the stable conformers all adopt boat conformation.^8,11^ We postulated two types of steric effect are responsible for the outcome: (1) steric effect from the phenyl ring; (2) steric effect from the hydrogen and the carbonyl (Fig. 3k). The distance *x* between carbonyl oxygen and hydrogen in the benzene ring were measured from the corresponding crystal structures and illustrated in Fig. 3. It is about 4.0 Å for the (*R*,*M*)-5b, (*S*,*P*)-5c and (*R*,*M*)-5d. This pocket may produce steric hindrance when bulky substituents are in the equatorial position.

As for 5d with a quaternary carbon, the axial methyl group of the minor conformer would affect the orientation of CH_3_ of the ethyl group and push it towards the carbonyl side as shown in Fig. 3k, the steric hindrance from the hydrogen and the carbonyl dominates making the ring higher in energy. When the ethyl group is on the axial position of the seven-membered ring in the major conformer, the steric effect from the hydrogen and the carbonyl is diminished. However, the steric hindrance from the phenyl ring is dominant, which is smaller than the steric hindrance from the hydrogen and the carbonyl in the minor conformer because of more space around (Fig. 3j). In such cases, the size or volume of the substituents can be used as a parameter to reflect the energy of the system. The volume in the “anchor sphere” (*V*^a^) for a CH_3_ substituent is 28.4 Å^3^ that is smaller than the ethyl group of 43.1 Å^3^.^32^ This should be responsible for the high ratio of 10 : 1 and the dominant conformer with axial ethyl group of the 5d experimentally observed. Similarly, 5c and 5b possess the substituents with increasing *V*^a^ of 47.8 Å^3^ and 52.6 Å^3^ that are more crowded for the pocket in the equatorial position,^32^ which leads to higher stereoselectivity and the stable conformation with larger groups on the axial bonds as well. Furthermore, the interaction region indicator (IRI) for 5d was conducted with the help of the Multiwfn program.^33,34^ The isosurface maps of two conformations of 5d (see Fig. S51 and S52†) indicate that the conformation with the larger ethyl group on the axial position exhibits larger areas of weak interactions with the benzene ring, enhancing its stability due to lower energy. On the other hand, the steric effect from the phenyl ring is less profound and when larger substituents are in the axial positions the steric tension is released. However, in the case of 5a with a tertiary carbon, the pocket is relatively sufficient to place the methyl and steric effect from the phenyl ring and the methyl plays a dominant role in this case and thereby the conformer with methyl group in the equatorial position is more stable.

## Conclusion

In summary, we have constructed a series of 7,7-disubstituted-dibenzo[*b*,*d*]oxepin-6(7*H*)-ones and studied their conformations. Single crystal diffraction studies indicate that the stable conformers all favor to place the larger groups in the axial positions instead of the equatorial positions. In this system, two types of steric effect are responsible for the preferential conformation and the steric hindrance from the hydrogen and the carbonyl is prominent. Furthermore, the size or volume of the substituents can be used as a parameter to rationalize the results and reflect the energy of the system. As we increase the size of substituents on the quaternary carbon, the stereoselectivity becomes higher. In addition, the system we established is highly sensitive to the size of substituents in the quaternary carbon center and even a 10 : 1 selectivity between two conformers is achieved with methyl and ethyl substituents. This study may provide information for cognition and understanding of the conformations of other cyclic systems with a quaternary center.

## Data availability

The data supporting this article have been included as part of the ESI.†

## Conflicts of interest

There are no conflicts to declare.

## Supplementary Material

RA-014-D4RA04703F-s001

RA-014-D4RA04703F-s002
