# Supplementary material for: Conformational studies of biaryl-bridged seven-membered lactones with a quaternary carbon center
Source: RSC Adv. 2024 Aug 14;14(35):25663–8. doi: 10.1039/d4ra04703f (PMC11324063; doi:10.1039/d4ra04703f)
Supplement: RA-014-D4RA04703F-s001 [file RA-014-D4RA04703F-s001.pdf]

Supplementary Information (SI) for RSC Advances.
This journal is © The Royal Society of Chemistry 2024

Supporting Information

Conformational studies of biaryl-bridged seven-membered lactones with a quaternary carbon center

Yu Zhang, Yueyang Yu, Xiaoqiang Ma, Xiaofei Zhao, Jialin Huang and Depeng Zhao*

Table of Contents

General remarks 3
Procedures for the synthesis of **5b-5e** 4
Procedures for the synthesis of **5f** 9
Crystallographic data of **5b-5d** 12
Low-temperature NMR experiments 15
Computational studies 16
NMR Spectra and HRMS spectra 28
IRI of **5d** 52
Reference 54

General remarks

Column chromatography was performed on silica gel (Silica-P flash silica gel from Silicycle, size 40-63 µm). TLC was performed on silica gel 60/ Kieselguhr F254. Flash column chromatography was performed on Biotage Isolelera One with prepacked columns. Mass spectra were recorded on a AEI-MS-902 mass spectrometer (EI+) or a LTQ Orbitrap XL (ESI+). ^{1}H, ^{13}C NMR were recorded on a Varian AMX400 (400 and 100.6 MHz, respectively) or a Varian Unity Plus Varian-500 (500 and 125 MHz, respectively). Chemical shift values for ^{1}H and ^{13}C NMR are reported in ppm with the solvent resonance as the internal standard ($CHCl_3$: δ 7.26 ppm for ^{1}H, δ 77.0 ppm for ^{13}C; DMSO: δ 2.50 ppm for ^{1}H, δ 39.52 ppm for ^{13}C). Data are reported as follows: chemical shifts, multiplicity (s = singlet, d = doublet, t = triplet, q = quartet, br = broad, m = multiplet), coupling constants (Hz), and integration. All reactions were performed under anhydrous conditions under N_2 atmosphere. All chemicals used were of analytical grade and were used as received without any further purification. All anhydrous solvents used in reactions were purchased in SureSeal bottles or dried over molecular sieves.

Procedures for the synthesis of 5b-5e

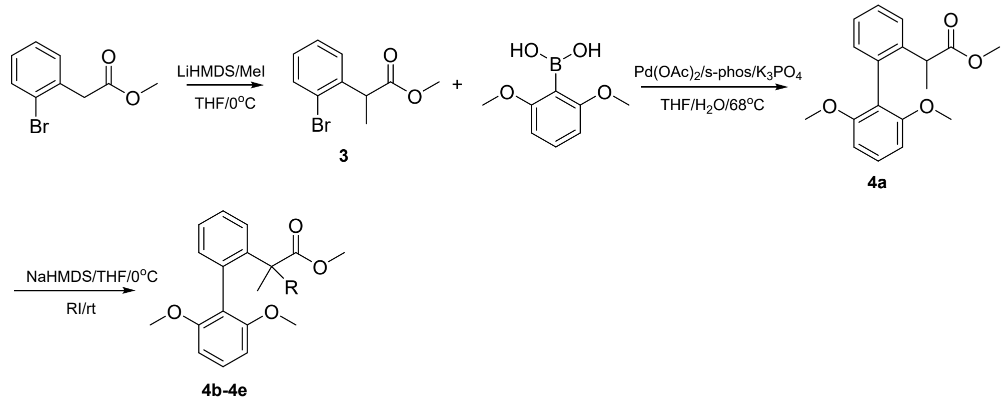

Scheme S1. The synthesis diagram of compound **4a**-**4e**.

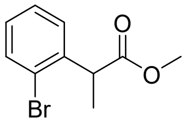

methyl 2-(2-bromophenyl) acetate

To a solution of methyl 2-(2-bromophenyl)acetate (5.0 g, 22.0 mmol) in THF (20.0 mL) at 0 °C was added a solution of LiHMDS (1.0 M in THF, 26.4 mL, 26.4 mmol). The mixture was stirred at 0 °C for 1 h, then was treated with iodomethane (2.1 mL, 33.0 mmol). The reaction mixture was warmed to room temperature and stirred for another 5 h. The mixture was quenched with saturated NH_4Cl, then was extracted with EtOAc. The combined organic extracts were washed with brine, dried (Na_2SO_4) and concentrated. The product was purified by flash chromatography (gradient elution, 2-5 % EtOAc in hexanes) to afford 3.5 g (70 % yield) of **3** as a colorless transparent liquid.

^{1}H NMR (500 MHz, $CDCl_3$) δ 7.56 (d, J = 8.2 Hz, 1H), 7.36 – 7.21 (m, 2H), 7.14 – 7.04 (m, 1H), 4.23 (q, J = 7.2 Hz, 1H), 3.68 (s, 3H), 1.49 (d, J = 7.2 Hz, 3H).

^{13}C NMR (126 MHz, $CDCl_3$) δ 174.6, 140.3, 133.1, 128.7, 128.5, 128.0, 124.4, 52.3, 44.7, 18.0.

HRMS (ESI+, m/z) calculated for $C_{10}H_{11}O_2Br$ $[M + H]^+$: 243.0015; found: 243.0060.

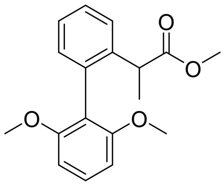

methyl 2-(2',6'-dimethoxy-[1,1'-biphenyl]-2-yl) propanoate

A 50.0 mL round bottom flask with stirring bar was charged with **3** (3.0 g, 12.4 mmol), 2,6-dimethoxyboricacid (3.4 g, 18.6 mmol), K_3PO_4 (3.4 g, 24.8 mmol), palladium(II)acetate (139.2 mg, 0.6 mmol) and 2-dicyclohexylphosphino-2',6'-dimethoxybiphenyl (763.5 mg, 1.9 mmol) and evacuated and back-filled with nitrogen (three times). Then degassed THF (10.0 mL) and H_2O (10.0 mL) were added successively. The reaction was stirred at 68 °C for 16 h, after which the reaction mixture was cooled to room temperature, followed by addition of water (20.0 mL). The organic and aqueous phases were then separated, and the aqueous phase was extracted with ethyl acetate (30.0 mL, three times). The combined organic phases were washed with brine (20.0 mL), dried over Na_2SO_4, and concentrated under reduced pressure. The residue was purified by flash chromatography on silica with pentane/ethyl acetate (10:1) to afford **4a** as a white solid (2.1 g, 70 % yield).

^{1}H NMR (400 MHz, $CDCl_3$) δ 7.48 (dd, J = 7.7, 1.1 Hz, 1H), 7.42 – 7.27 (m, 3H), 7.16 (dd, J = 7.5, 1.4

Hz, 1H), 6.67 (dd, *J* = 8.4, 3.3 Hz, 2H), 3.72 (s, 3H), 3.71 (s, 3H), 3.60 (s, 3H), 3.55 (q, *J* = 7.2 Hz, 1H), 1.38 (d, *J* = 7.2Hz, 3H).

^{13}C NMR (101 MHz, $CDCl_3$) δ 175.7, 158.2, 157.9, 140.0, 134.0, 131.1, 129.2, 127.8, 126.7, 126.6, 118.1, 104.1, 103.8, 55.8, 55.7, 51.8, 41.9, 19.0

HRMS (ESI+, m/z) calculated for $C_{18}H_{20}O_4$ [M + Na]$^+$: 323.1254; found: 323.1266.

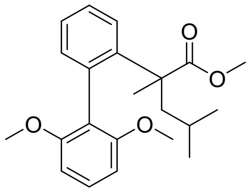

methyl 2-(2',6'-dimethoxy-[1,1'-biphenyl]-2-yl)-2,4-dimethylpentanoate

To a solution of **4a** (1.0 g, 3.3 mmol) in THF (20.0 mL) at 0 °C was added a solution of NaHMDS (1.0 M in THF, 6.6 mL, 6.6 mmol). The mixture was stirred at 0 °C for 1 h, then was treated with isobutyl iodide (949.4 μL, 8.3 mmol). The reaction mixture was warmed to room temperature and stirred overnight. The mixture was quenched with saturated NH_4Cl, then was extracted with ethyl acetate. The combined organic extracts were washed with brine, dried (Na_2SO_4) and concentrated. The product was purified by flash chromatography to afford 0.7 g (70 % yield) of **4b** as a colorless liquid.

^{1}H NMR (400 MHz, $CDCl_3$) δ 7.44 (d, *J* = 7.9 Hz, 1H), 7.38 – 7.22 (m, 3H), 7.00 (dd, *J* = 7.4, 1.3 Hz, 1H), 6.62 (dd, *J* = 8.3, 2.2 Hz, 2H), 3.74 (s, 6H), 3.42 (s, 3H), 1.91 (ddd, *J* = 67.9, 13.7, 5.3 Hz, 2H), 1.62 – 1.50 (m, 1H), 1.41 (d, *J* = 9.6 Hz, 3H), 0.91 – 0.80 (m, 6H).

^{13}C NMR (101 MHz, $CDCl_3$) δ 176.7, 158.1, 143.9, 133.2, 133.1, 129.1, 127.7, 127.4, 126.3, 120.2, 103.6, 103.5, 55.7, 55.5, 51.9, 51.5, 48.0, 25.2, 25.1, 24.6, 23.7.

HRMS (ESI+, m/z) calculated for $C_{22}H_{28}O_4$ [M + Na]$^+$: 379.1880; found: 379.1894.

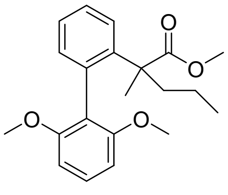

methyl 2-(2',6'-dimethoxy-[1,1'-biphenyl]-2-yl)-2-methylpentanoate

To a solution of **4a** (1.0 g, 3.3 mmol) in THF (20.0 mL) at 0 °C was added a solution of NaHMDS (1.0 M in THF, 6.6 mL, 6.6 mmol). The mixture was stirred at 0 °C for 1 h, then was treated with 1-iodopropane (804.6 μL, 8.3 mmol). The reaction mixture was warmed to room temperature and stirred overnight. The mixture was quenched with saturated NH_4Cl, then was extracted with ethyl acetate. The combined organic extracts were washed with brine, dried (Na_2SO_4) and concentrated. The product was purified by flash chromatography (gradient elution, 2-5 % EtOAc in hexanes) to afford 0.8 g (75 % yield) of **4c** as a colorless transparent liquid.

^{1}H NMR (400 MHz, $CDCl_3$) δ 7.48 – 7.24 (m, 1H), 7.01 (dd, *J* = 7.4, 1.3 Hz, 1H), 6.61 (dd, *J* = 8.3, 2.6 Hz, 1H), 3.71 (d, *J* = 0.8 Hz, 2H), 3.40 (s, 1H), 1.91 – 1.75 (m, 1H), 1.42 (s, 1H), 1.26 – 1.00 (m, 1H), 0.87 (t, *J* = 7.3 Hz, 1H).

^{13}C NMR (126 MHz, $CDCl_3$) δ 176.5, 158.3, 158.2, 143.0, 133.5, 133.0, 129.1, 127.8, 127.2, 126.3, 119.9, 103.6, 103.6, 55.6, 55.5, 51.5, 51.3, 41.5, 24.2, 18.1, 14.7.

HRMS (ESI+, m/z) calculated for $C_{21}H_{26}O_4$ [M + Na]$^+$: 365.1723; found: 365.1738.

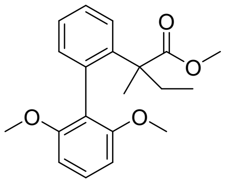

methyl 2-(2',6'-dimethoxy-[1,1'-biphenyl]-2-yl)-2-methylbutanoate

To a solution of **4a** (1.0 g, 3.3 mmol) in THF (20.0 mL) at 0 °C was added a solution of NaHMDS (1.0 M in THF, 6.6 mL, 6.6 mmol). The mixture was stirred at 0 °C for 1 h, then was treated with ethyl iodide (660.0 μL, 8.3 mmol). The reaction mixture was warmed to room temperature and stirred overnight. The mixture was quenched with saturated NH_4Cl, then was extracted with ethyl acetate. The combined organic extracts were washed with brine, dried (Na_2SO_4) and concentrated. The product was purified by flash chromatography (gradient elution, 2-5 % EtOAc in hexanes) to afford 0.8 g (80 % yield) of **4d** as a colorless transparent liquid.

^{1}H NMR (400 MHz, $CDCl_3$) δ 7.47 – 7.21 (m, 2H), 7.02 (d, *J* = 7.4 Hz, 1H), 6.62 (d, *J* = 8.4 Hz, 1H), 3.72 (d, *J* = 2.2 Hz, 3H), 3.41 (s, 1H), 2.00 – 1.82 (m, 1H), 1.41 (s, 1H), 0.76 (t, *J* = 7.4 Hz, 1H).

^{13}C NMR (101 MHz, $CDCl_3$) δ 176.5, 158.3, 158.2, 142.7, 133.6, 132.9, 129.2, 127.9, 127.3, 126.4, 119.8, 103.6, 103.6, 76.8, 55.6, 55.5, 51.6, 31.6, 23.7, 9.3.

HRMS (ESI+, m/z) calculated for $C_{20}H_{24}O_4$ $[M + Na]^+$: 351.1567; found: 351.1581.

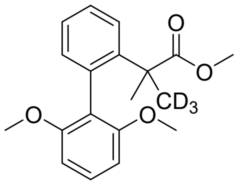

methyl 2-(2',6'-dimethoxy-[1,1'-biphenyl]-2-yl)-2-methylpropanoate-3,3,3-d$_3$

To a solution of **4a** (1.0 g, 3.3 mmol) in THF (20.0 mL) at 0 °C was added a solution of NaHMDS (1.0 M in THF, 6.6 mL, 6.6 mmol). The mixture was stirred at 0 °C for 1 h, then was treated with iodomethane-d$_3$ (513.3 μL, 8.3 mmol). The reaction mixture was warmed to room temperature and stirred overnight. The mixture was quenched with saturated NH_4Cl, then was extracted with ethyl acetate. The combined organic extracts were washed with brine, dried (Na_2SO_4) and concentrated. The product was purified by flash chromatography to afford 0.7 g (70 % yield) of **4e** as a colorless liquid.

^{1}H NMR (400 MHz, $CDCl_3$) δ 7.48 (dd, *J* = 8.0, 0.9 Hz, 1H), 7.41 – 7.27 (m, 3H), 7.04 (dd, *J* = 7.5, 1.5 Hz, 1H), 6.64 (d, *J* = 8.4 Hz, 2H), 3.73 (s, 6H), 3.44 (s, 3H), 1.47 (s, 3H).

^{13}C NMR (101 MHz, $CDCl_3$) δ 177.2, 158.1, 143.4, 133.3, 132.8, 129.1, 127.5, 127.0, 126.4, 119.8, 103.6, 55.5, 51.7, 47.3, 27.4.

HRMS (ESI+, m/z) calculated for $C_{19}H_{19}D_3O_4$ $[M + Na]^+$: 340.1599; found: 340.1617.

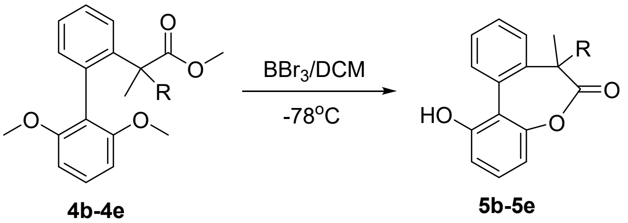

Scheme S2. The synthesis diagram of compound **5b-5e**.

BBr_3 (6.0 eq) was added to a stirred solution of **4b-4e** in dry dichloromethane in a flame-dried round bottom flask at -78 °C under N_2 atmosphere and the reaction mixture was stirred overnight. Then the

reaction was allowed to warm to room temperature and stirred for another 30 min. The reaction was quenched with ice/H_2O at 0 °C and the mixture extracted with ethyl acetate (3×30 mL). The combined organic layers were dried over Na_2SO_4 and evaporated under reduced pressure. The residue was purified by flash chromatography on silica with pentane/ethyl acetate (4:1) to afford **5b-5e**. (85% yield).

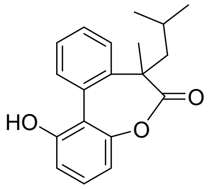

1-hydroxy-7-isobutyl-7-methyldibenzo[b,d]oxepin-6(7H)-one

White solid (yield: 75 %), m.p.= 82°C

^{1}H NMR (400 MHz, $CDCl_3$) δ 7.74 – 7.67 (m, 1H), 7.67 – 7.59 (m, 1H), 7.51 – 7.40 (m, 2H), 7.28 (t, *J* = 8.0 Hz, 1H), 6.88 (d, *J* = 8.1 Hz, 1H), 6.84 (d, *J* = 8.1 Hz, 1H), 5.62 (s, 1H), 1.87 (s, 3H), 1.59 – 1.48 (m, 1H), 1.47 – 1.33 (m, 2H), 0.65 (d, *J* = 6.6 Hz, 3H), 0.55 (d, *J* = 6.5 Hz, 3H).

^{13}C NMR (126 MHz, $CDCl_3$) δ 172.9, 152.7, 150.9, 140.8, 130.3, 129.9, 129.3, 128.2, 127.6, 118.7, 113.1, 112.3, 51.8, 42.6, 25.9, 24.4, 24.1, 22.9.

HRMS (ESI+, m/z) calculated for $C_{19}H_{20}O_3$ $[M + H]^+$: 297.1485; found: 297.1503.

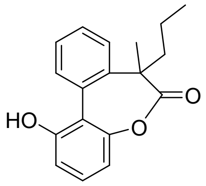

White solid (yield: 73%), m.p.= 147°C

1-hydroxy-7-methyl-7-propyldibenzo[b,d]oxepin-6(7H)-one

^{1}H NMR (400 MHz, $CDCl_3$) δ 7.78 – 7.69 (m, 1H), 7.66 – 7.58 (m, 1H), 7.49 – 7.40 (m, 2H), 7.26 (t, *J* = 6.0 Hz, 1H), 6.88 (d, *J* = 8.1 Hz, 1H), 6.83 (d, *J* = 8.1 Hz, 1H), 5.86 (s, 1H), 1.81 (s, 3H), 1.50 – 1.35 (m, 1H), 1.35 – 1.15 (m, 2H), 1.08 – 0.94 (m, 1H), 0.55 (t, *J* = 7.2 Hz, 3H).

^{13}C NMR (101 MHz, $CDCl_3$) δ 172.9, 152.6, 150.5, 140.3, 130.2, 130.1, 129.7, 129.2, 128.1, 127.5, 118.6, 113.2, 112.1, 52.2, 37.0, 24.0, 19.4, 14.3.

HRMS (ESI+, m/z) calculated for $C_{18}H_{18}O_3$ $[M + H]^+$: 283.1329; found: 283.1323.

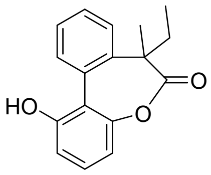

White solid (yield: 77 %), m.p.= 156°C

7-ethyl-1-hydroxy-7-methyldibenzo[b,d]oxepin-6(7H)-one

^{1}H NMR (500 MHz, $CDCl_3$) δ 7.80 – 7.74 (m, 1H), 7.66 – 7.62 (m, 1H), 7.49 – 7.44 (m, 2H), 7.31 – 7.20 (m, 1H), 6.90 (d, *J* = 8.2 Hz, 1H), 6.86 (d, *J* = 8.2 Hz, 1H), 6.01 (s, 1H), 1.82 (s, 3H), 1.62 – 1.53 (m, 1H), 1.46 – 1.36 (m, 1H), 0.72 (t, *J* = 7.4 Hz, 3H).

^{13}C NMR (126 MHz, $CDCl_3$) δ 173.0, 152.7, 150.4, 140.0, 130.2, 129.7, 129.2, 128.1, 127.6, 118.5, 113.3, 112.0, 52.6, 27.6, 23.3, 10.2.

HRMS (ESI+, m/z) calculated for $C_{18}H_{18}O_3$ $[M + H]^+$: 267.1027; found: 267.1020.

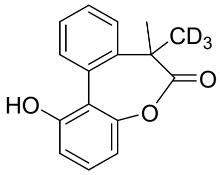

White solid (yield: 80 %), m.p.= 198°C

1-hydroxy-7-methyl-7-(methyl-d$_3$) dibenzo[b,d]oxepin-6(7H)-one

^{1}H NMR (500 MHz, $CDCl_3$) δ 7.77 – 7.72 (m, 1H), 7.62 (dq, *J* = 4.0, 7.9 Hz, 1H), 7.50 – 7.42 (m, 2H), 7.26 (t, *J* = 8.2 Hz, 1H), 6.86 (dd, *J* = 8.1, 16.8 Hz, 2H), 1.81 (s, 2H), 1.14 (s, 2H).

^{13}C NMR (126 MHz, $CDCl_3$) δ 172.9, 152.6, 150.8, 140.9, 130.0, 129.9, 129.8, 129.1, 128.1, 126.8, 118.3, 113.0, 112.0, 46.8, 26.6, 22.1.

HRMS (ESI+, m/z) calculated for $C_{16}H_{11}D_3O_3$ [M - H]$^-$: 256.1058; found: 256.1060.

Procedures for the synthesis of 5f

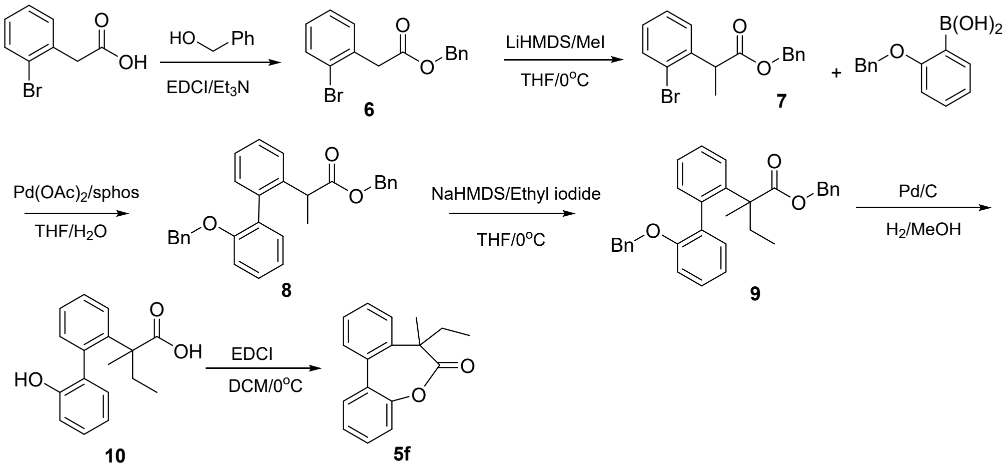

Scheme S3. The synthesis diagram of compound **5f**.

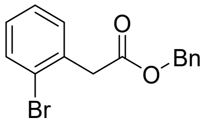

benzyl 2-(2-bromophenyl) acetate

To a solution of 2-(2-bromophenyl) acetic acid (3.0 g, 14.0 mmol) and benzyl alcohol (1.5 mL, 14.0 mmol) in DCM (20.0 mL) were added EDCI (1-ethyl-3-(3-dimethylaminopropyl) carbodiimide) (4.0 g, 21.0 mmol) and trimethylamine (2.9 mL, 21.0 mmol). The mixture was stirred at room temperature overnight and was quenched with saturated NH_4Cl, then was extracted with ethyl acetate. The combined organic extracts were washed with brine, dried (Na_2SO_4) and concentrated. The product was purified by flash chromatography to afford 2.8 g (92 % yield) of **6** as a colorless liquid.

^{1}H NMR (500 MHz, $CDCl_3$) δ 7.45 (d, *J* = 7.9 Hz, 1H), 7.27 – 7.10 (m, 7H), 7.05 – 6.97 (m, 1H), 5.06 (s, 2H), 3.73 (s, 2H).

^{13}C NMR (126 MHz, $CDCl_3$) δ 170.4, 135.8, 134.2, 132.9, 131.6, 129.0, 128.6, 128.3, 128.2, 127.6, 125.1, 66.8, 41.7.

HRMS (ESI+, m/z) calculated for $C_{15}H_{14}O_2Br$ $[M + Na]^+$: 328.0069; found: 328.0029.

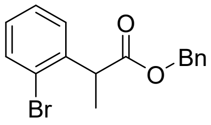

benzyl 2-(2-bromophenyl) propanoate

Lithium bis(trimethylsilyl)amide (1.0 M in THF, 6.9 mL, 6.9 mmol) was added to a stirred solution of **6** (2.0 g, 6.6 mmol) in dry THF in a flame-dried round bottom flask at 0 °C under N_2 atmosphere and the reaction mixture was stirred for 1h at 0 °C, then was treated with iodomethane (429.8 μL,6.9 mmol). The reaction mixture was warmed to room temperature and stirred overnight. The mixture was quenched with saturated NH_4Cl and was extracted with ethyl acetate. The combined organic extracts were washed with brine, dried (Na_2SO_4) and concentrated. The product was purified by flash chromatography to afford 1.7 g (85 % yield) of **7** as a colorless liquid.

^{1}H NMR (500 MHz, $CDCl_3$) δ 7.60 (d, *J* = 8.0 Hz, 1H), 7.39 – 7.24 (m, 7H), 7.18 – 7.09 (m, 1H), 5.26 – 5.08 (m, 2H), 4.32 (q, *J* = 7.2 Hz, 1H), 1.54 (d, *J* = 7.2 Hz, 3H).

^{13}C NMR (126 MHz, $CDCl_3$) δ 173.9, 140.1, 136.0, 133.1, 128.7, 128.6, 128.5, 128.2, 128.0, 127.9, 124.6, 66.7, 44.8, 17.9.

HRMS (ESI+, m/z) calculated for $C_{16}H_{15}O_2Br$ [M + Na]$^+$: 341.0148; found: 341.0171.

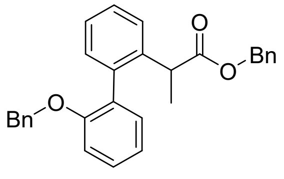

benzyl 2-(2'-(benzyloxy)-[1,1'-biphenyl]-2-yl) propanoate

A 100.0 mL round bottom flask with stirring bar was charged with **7** (1.7 g, 5.3 mmol), (2-(benzyloxy) phenyl)boronic acid (1.8 g, 8.0 mmol), K_3PO_4 (2.3 g, 10.6 mmol), palladium(II)acetate (59.5 mg, 0.3 mmol) and 2-dicyclohexylphosphino-2',6'-dimethoxybiphenyl (363.3 mg, 0.9 mmol) and evacuated and back-filled with nitrogen (three times). Then degassed THF (20.0 mL) and H_2O (20.0 mL) were added successively. The reaction was stirred at 65 °C overnight, after which the reaction mixture was cooled to room temperature, followed by addition of water (50.0 mL). The organic and aqueous phases were then separated and the aqueous phase was extracted with ethyl acetate (30.0 mL, three times). The combined organic phases were washed with brine (50.0 mL), dried over Na_2SO_4, and concentrated under reduced pressure. The residue was purified by flash chromatography on silica with pentane/ethyl acetate (10:1) to afford **8** as a mixture of atropisomers (1.3 g, 78 % yield).

NMR spectra was acquired with a mixture of atropisomers **8**.

^{1}H NMR (500 MHz, $CDCl_3$) δ 7.40 – 6.83 (m, 18H), 5.09 – 4.67 (m, 4H), 3.76 – 3.62 (m, 1H), 1.36 – 1.23 (m, 3H).

^{13}C NMR (126 MHz, $CDCl_3$) δ 175.0, 174.9, 156.0, 155.8, 139.6, 139.4, 138.6, 138.2, 137.5, 137.3, 136.3, 136.3, 132.1, 131.2, 130.8, 130.7, 130.6, 129.0, 128.9, 128.5, 128.5, 128.4, 128.4, 128.0, 127.9, 127.9, 127.7, 127.7, 127.4, 127.0, 126.7, 126.7, 126.6, 126.5, 126.4, 121.1, 121.0, 113.6, 113.0, 70.3, 70.2, 66.2, 66.1, 42.0, 41.8, 20.0, 18.2.

HRMS (ESI+, m/z) calculated for $C_{29}H_{26}O_3$ [M + Na]$^+$: 445.1774; found: 445.1817.

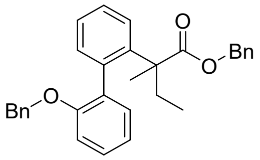

benzyl 2-(2'-(benzyloxy)-[1,1'-biphenyl]-2-yl)-2-methylbutanoate

Sodium bis(trimethylsilyl)amide (1.0 M in THF, 4.7 mL, 4.7 mmol) was added to a stirred solution of **8** (1.0 g, 2.4 mmol) in dry THF in a flame-dried round bottom flask at 0 °C under N_2 atmosphere and the reaction mixture was stirred for 1 h at 0 °C, then was treated with iodoethane (376.0 μL,4.7 mmol). The reaction mixture was warmed to room temperature and stirred overnight. The mixture was quenched with saturated NH_4Cl and was extracted with ethyl acetate. The combined organic extracts were washed with brine, dried (Na_2SO_4) and concentrated. The product was purified by flash chromatography to afford 900.0 mg (90 % yield) of **9** as a colorless liquid.

NMR spectra was acquired with a mixture of atropisomers **9**.

^{1}H NMR (400 MHz, $CDCl_3$) δ 7.46 (dd, J = 1.4, 8.1 Hz, 1H), 7.41 (dd, J = 1.5, 8.0 Hz, 1H), 7.34 (tt, J = 1.6, 7.9 Hz, 2H), 7.30 – 7.20 (m, 20H), 7.19 – 7.12 (m, 8H), 7.09 (ddd, J = 1.7, 7.5, 12.3 Hz, 5H), 6.98 – 6.88 (m, 5H), 5.07 – 4.93 (m, 5H), 4.89 – 4.74 (m, 5H), 2.09 – 1.95 (m, 2H), 1.87 (dt, J = 7.4, 14.9 Hz, 1H), 1.75 – 1.65 (m, 1H), 1.49 (s, 4H), 1.32 (s, 3H), 0.74 (t, J = 7.4 Hz, 3H), 0.51 (t, J = 7.4 Hz, 4H).

^{13}C NMR (101 MHz, $CDCl_3$) δ 176.7, 175.9, 156.4, 156.1, 142.5, 141.0, 137.9, 137.7, 137.4, 137.3, 136.2, 136.1, 132.5, 132.4, 132.0, 131.8, 131.7, 131.4, 128.8, 128.3, 128.3, 128.2, 127.9, 127.7, 127.5,

127.3, 127.2, 127.0, 126.8, 126.4, 126.4, 126.0, 125.9, 119.9, 119.8, 112.3, 112.1, 69.6, 69.6, 65.9, 50.9, 50.8, 32.4, 30.1, 24.4, 23.2, 9.0, 8.7.

HRMS (ESI+, m/z) calculated for $C_{31}H_{30}O_3$ $[M + Na]^+$: 473.2087; found: 473.2094.

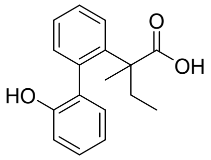

2-(2'-hydroxy-[1,1'-biphenyl]-2-yl)-2-methylbutanoic acid

To a solution of **9** (800.0 mg, 1.8 mmol) in MeOH (20.0 mL) was added Pd/C (300.0 mg, 10% on Carbon wetted with ca. 55% water). The mixture was stirred under H_2 atmosphere at room temperature overnight until it was completed judged by TLC and was quenched with saturated NH_4Cl. After this time Pd/C was filtered off and was extracted with ethyl acetate. The combined organic extracts were washed with brine, dried (Na_2SO_4) and concentrated. The product was purified by flash chromatography to afford 760.0 mg (92 % yield) of **10** as a colorless liquid.

NMR spectra was acquired with a mixture of atropisomers **10**.

^{1}H NMR (500 MHz, $CDCl_3$) δ 7.53 (dd, *J* = 12.2, 8.1 Hz, 1H), 7.43 (tdd, *J* = 8.1, 3.6, 1.5 Hz, 1H), 7.33 (td, *J* = 7.4, 0.9 Hz, 1H), 7.22 (td, *J* = 7.7, 0.7 Hz, 1H), 7.17 – 7.11 (m, 1.5H), 7.07 (dd, *J* = 7.5, 1.5 Hz, 0.5H), 6.89 – 6.81 (m, 2H), 2.09 – 1.93 (m, 1.5H), 1.87 (dq, *J* = 14.7, 7.4 Hz, 0.5H), 1.56 (s, 1.5H), 1.49 (s, 1.5H), 0.91 – 0.83 (m, 1.5H), 0.78 (t, *J* = 7.5 Hz, 1.5H).

^{13}C NMR (126 MHz, $CDCl_3$) δ 182.7, 182.3, 153.4, 153.2, 143.4, 142.3, 135.5, 135.4, 133.0, 133.0, 131.4, 131.2, 129.8, 129.7, 128.7, 128.4, 128.2, 128.1, 127.9, 127.8, 127.3, 127.2, 119.7, 119.6, 115.8, 115.7, 51.0, 50.6, 31.8, 31.0, 24.7, 24.1, 9.1, 8.9.

HRMS (ESI+, m/z) calculated for $C_{17}H_{18}O_3$ $[M + Na]^+$: 293.1203; found: 293.1148.

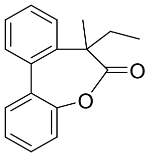

7-ethyl-7-methyldibenzo[b,d]oxepin-6(7H)-one

To a solution of **10** (500.0 mg, 1.9 mmol) in DCM (20.0 mL) was added EDCI (1-ethyl-3-(3-dimethylaminopropyl) carbodiimide) (710.0 mg, 3.7 mmol). The mixture was stirred at 0 °C for 30 min and was quenched with saturated NH_4Cl, then was extracted with ethyl acetate. The combined organic extracts were washed with brine, dried (Na_2SO_4) and concentrated. The product was purified by flash chromatography to afford 450.0 mg (90 % yield) of **5f** as a colorless liquid.

^{1}H NMR (400 MHz, $CDCl_3$) δ 7.64 – 7.58 (m, 1H), 7.58 – 7.50 (m, 2H), 7.49 – 7.35 (m, 3H), 7.29 (td, *J* = 7.6, 1.2 Hz, 1H), 7.25 – 7.19 (m, 1H), 1.76 (s, 3H), 1.61 – 1.48 (m, 1H), 1.44 – 1.33 (m, 1H), 0.72 (t, *J* = 7.3 Hz, 3H).

^{13}C NMR (126 MHz, $CDCl_3$) δ 172.1, 149.7, 139.0, 135.4, 131.1, 130.1, 129.7, 129.4, 128.9, 128.4, 126.5, 125.5, 119.6, 52.3, 28.0, 22.9, 10.1.

HRMS (ESI+, m/z) calculated for $C_{17}H_{16}O_2$ $[M + Na]^+$: 275.1043; found: 275.1088.

Crystallographic data of 5b-5d

The computational formula of R_1 and ωR_2 are shown below[1] (F represents the structure factor, the subscripts "o" and "c" respectively denote the observed and calculated values):

$$R_1 = \frac{\sum ||F_o| - |F_c||}{\sum |F_o|}$$

$$\omega R_2 = \left[\frac{\sum \omega({F_o}^2 - {F_c}^2)^2}{\sum \omega {F_o}^2}\right]^{1/2}$$

Identification code	zhangy_231123_auto
Empirical formula	$C_{19}H_{20}O_3$
Formula weight	296.35
Temperature/K	100.00(10)
Crystal system	monoclinic
Space group	$P2_1/n$
a/Å	8.3390(4)
b/Å	11.1632(6)
c/Å	16.2872(7)
α/°	90
β/°	93.877(4)
γ/°	90
Volume/Å^3	1512.70(13)
Z	4
ρ_{calc}mg/mm^3	1.301
μ/mm^{-1}	0.696
F(000)	632.0
Crystal size/mm^3	0.17 × 0.15 × 0.12
2Θ range for data collection	9.612 to 151.004°
Index ranges	-7 ≤ h ≤ 10, -13 ≤ k ≤ 13, -20 ≤ l ≤ 20
Reflections collected	8450
Independent reflections	2915[R(int) = 0.0630]
Data/restraints/parameters	2915/0/203
Goodness-of-fit on F^2	1.249
Final R indexes [I>=2σ (I)]	R_1 = 0.1090, ωR_2 = 0.3220
Final R indexes [all data]	R_1 = 0.1159, ωR_2 = 0.3254
Largest diff. peak/hole / e Å^{-3}	0.62/-0.42

Table S1. Crystal data and structure refinement for **5b**.

Crystal Data for $C_{19}H_{20}O_3$ (M =296.35 g/mol): monoclinic, space group $P2_1/n$ (no. 14), a = 8.3390(4) Å, b = 11.1632(6) Å, c = 16.2872(7) Å, β = 93.877(4)°, V = 1512.70(13) Å^3, Z = 4, T = 100.00(10) K, μ(Cu Kα) = 0.696 mm^{-1}, $Dcalc$ = 1.301 g/cm^3, 8450 reflections measured (9.612° ≤ 2Θ ≤ 151.004°), 2915 unique (R_{int} = 0.0630, R_{sigma} = 0.0558) which were used in all calculations. The final R_1 was 0.1090 (I > 2σ(I)) and wR_2 was 0.3254 (all data).

Identification code	zhangyu_181205
Empirical formula	$C_{18}H_{18}O_3$
Formula weight	282.32
Temperature/K	100.00(10)
Crystal system	orthorhombic
Space group	P212121
a/Å	7.96310(10)
b/Å	9.04150(10)
c/Å	19.89230(10)
α/°	90
β/°	90
γ/°	90
Volume/Å3	1432.21(3)
Z	4
ρcalcg/cm3	1.309
μ/mm-1	0.710
F(000)	600.0
Crystal size/mm3	0.3 × 0.2 × 0.1
Radiation	CuKα (λ = 1.54184)
2Θ range for data collection/°	8.89 to 154.016
Index ranges	-9 ≤ h ≤ 9, -11 ≤ k ≤ 10, -25 ≤ l ≤ 25
Reflections collected	14434
Independent reflections	2939 [Rint = 0.0259, Rsigma = 0.0168]
Data/restraints/parameters	2939/0/193
Goodness-of-fit on F2	1.049
Final R indexes [I>=2σ (I)]	R_1 = 0.0281, ωR_2 = 0.0741
Final R indexes [all data]	R_1 = 0.0289, ωR_2 = 0.0745
Largest diff. peak/hole / e Å-3	0.19/-0.17
Flack parameter	0.14(5)

Table S2. Crystal data and structure refinement for **5c**.

Crystal Data for $C_{18}H_{18}O_3$ (M =282.32 g/mol): orthorhombic, space group $P2_12_12_1$ (no. 19), a = 7.96310(10) Å, b = 9.04150(10) Å, c = 19.89230(10) Å, V = 1432.21(3) Å^3, Z = 4, T = 100.00(10) K, μ(CuKα) = 0.710 mm^{-1}, *Dcalc* = 1.309 g/cm^3, 14434 reflections measured (8.89° ≤ 2Θ ≤ 154.016°), 2939 unique (R_{int} = 0.0259, R_{sigma} = 0.0168) which were used in all calculations. The final R_1 was 0.0281 (I > 2σ(I)) and ωR_2 was 0.0745 (all data).

Identification code	zhangyu_181113
Empirical formula	$C_{17}H_{16}O_3$
Formula weight	268.30
Temperature/K	99.97(13)
Crystal system	orthorhombic
Space group	P212121
a/Å	9.0416(3)
b/Å	20.2173(5)
c/Å	22.2247(6)
α/°	90
β/°	90
γ/°	90
Volume/Å3	4062.6(2)
Z	12
ρcalcg/cm3	1.316
μ/mm-1	0.724
F(000)	1704.0
Crystal size/mm3	0.3 × 0.2 × 0.1
Radiation	CuKα (λ = 1.54184)
2Θ range for data collection/°	5.91 to 144.186
Index ranges	-11 ≤ h ≤ 9, -24 ≤ k ≤ 22, -26 ≤ l ≤ 27
Reflections collected	32061
Independent reflections	7974 [Rint = 0.1535, Rsigma = 0.0587]
Data/restraints/parameters	7974/0/550
Goodness-of-fit on F2	1.139
Final R indexes [I>=2σ (I)]	R_1 = 0.0734, ωR_2 = 0.1726
Final R indexes [all data]	R_1 = 0.0881, ωR_2 = 0.2089
Largest diff. peak/hole / e Å-3	0.40/-0.49
Flack parameter	0.14(15)

Table S3. Crystal data and structure refinement for **5d**.

Crystal Data for $C_{17}H_{16}O_3$ (*M* =268.30 g/mol): orthorhombic, space group $P2_12_12_1$ (no. 19), *a* = 9.0416(3) Å, *b* = 20.2173(5) Å, *c* = 22.2247(6) Å, *V* = 4062.6(2) Å^3, *Z* = 12, *T* = 99.97(13) K, μ(CuKα) = 0.724 mm^{-1}, *Dcalc* = 1.316 g/cm^3, 32061 reflections measured (5.91° ≤ 2Θ ≤ 144.186°), 7974 unique (R_{int} = 0.1535, R_{sigma} = 0.0587) which were used in all calculations. The final R_1 was 0.0734 (I > 2σ(I)) and ωR_2 was 0.2089 (all data).

Low-temperature NMR experiments

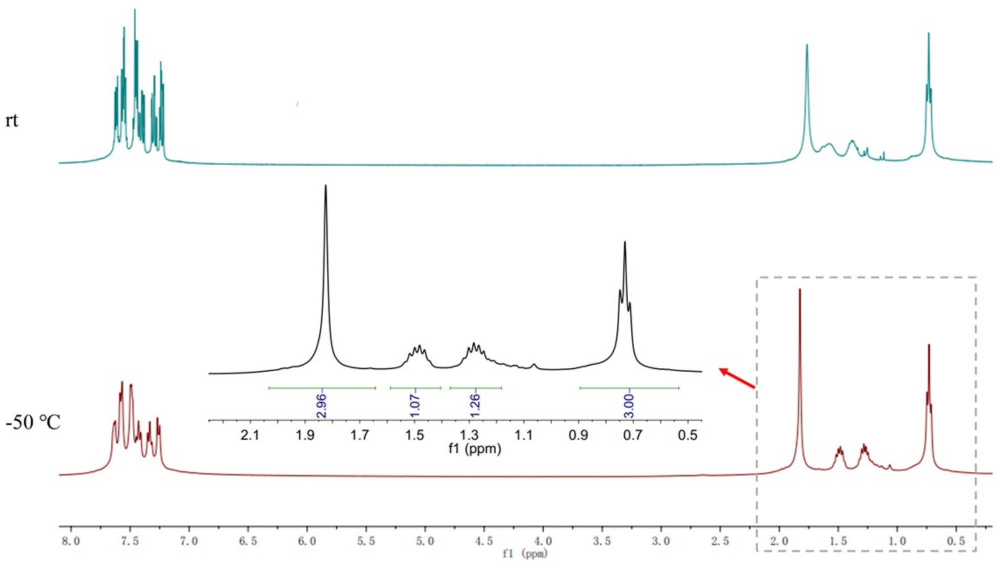

Figure S1. ^{1}H NMR spectra (400 MHz) of **5f** in $CDCl_3$.

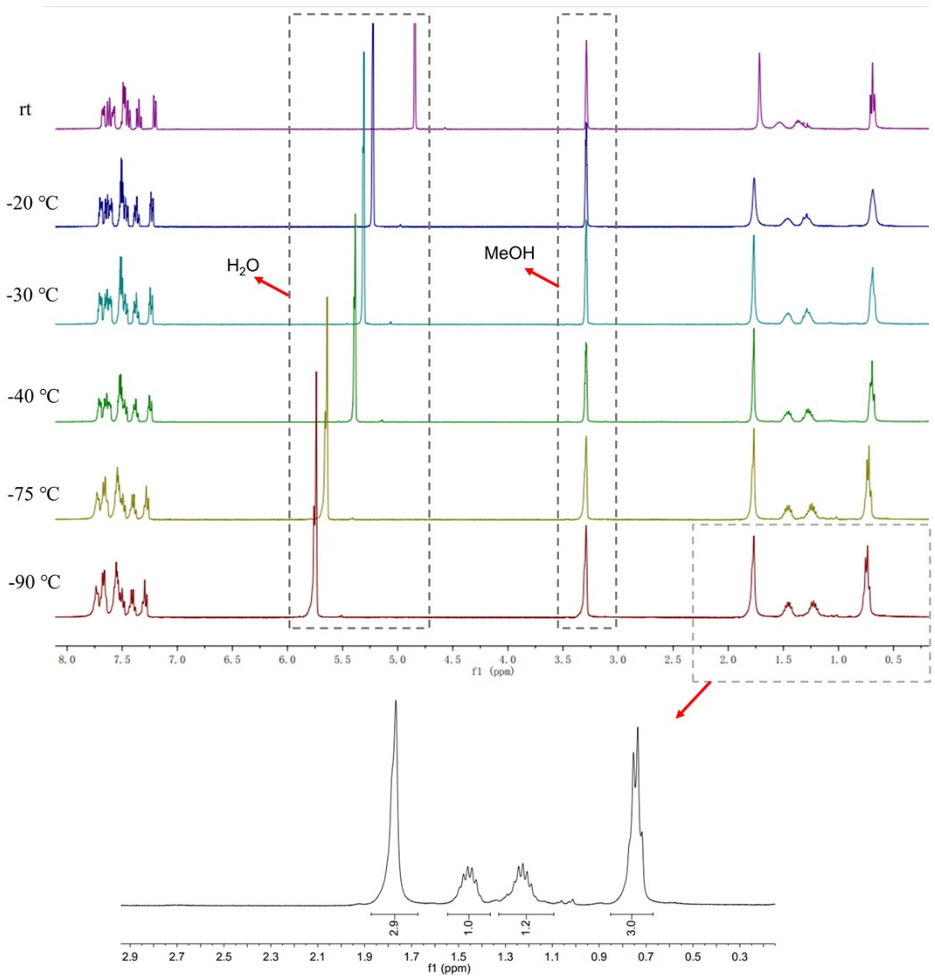

Figure S2. ^{1}H NMR spectra (400 MHz) of **5f** in CD_3OD.

Computational studies

All the calculations were carried out using the Gaussian16, Revision A.03 at the SMD(chloroform)-ωB97X-D/def2-TZVP level of theory.[2] The frequency analysis was calculated at the same level of theory to validate each structure as either a minimum or a transition state.[3] For transition state, the intrinsic reaction coordinate (IRC) analysis was conducted to ensure that it connects the right reactant and product.

(*R*,*M*)-**5b**	Coordinates (Angstroms)		
	X	Y	Z
O	0.590664	0.115468	1.902970
O	-2.083741	2.372464	-1.347840
1	-2.135933	3.234991	-1.771252
O	1.131910	-1.952224	2.257958
6	-1.632123	-0.035586	0.047875
6	0.239884	1.216113	1.158480
6	2.752252	-0.768763	-1.205720
1	3.016464	-1.805959	-1.427722
6	-3.023731	0.072522	0.022223
1	-3.475938	1.038938	0.196031
6	0.788000	-1.143016	1.444169
6	0.997505	2.350260	1.398115
1	1.809129	2.301110	2.110797
6	-0.837519	1.192927	0.279696
6	1.226765	-0.651362	-1.104055
1	0.795364	-0.978927	-2.054130
1	0.986565	0.405130	-1.019261
6	0.487604	-1.492510	-0.023782
6	0.698577	3.509723	0.709709
1	1.281850	4.405721	0.878690
6	-0.338216	3.525956	-0.206608
1	-0.563452	4.426037	-0.767359
6	-3.830069	-1.022920	-0.214727
1	-4.906974	-0.911224	-0.224985
6	-1.036721	-1.295138	-0.138083
6	-1.868351	-2.385666	-0.388542
1	-1.441395	-3.364280	-0.546887
6	-1.095525	2.382401	-0.423125
6	0.899858	-2.954506	-0.211119
1	1.962704	-3.071020	-0.009196
1	0.712386	-3.265434	-1.239960
1	0.372872	-3.623314	0.465416
6	-3.245134	-2.257668	-0.438600

1	-3.856942	-3.128281	-0.639914
6	3.474674	-0.353855	0.071978
1	3.184746	0.657550	0.371448
1	4.555942	-0.356580	-0.082832
1	3.269357	-1.027692	0.905306
6	3.223478	0.086244	-2.379815
1	2.724924	-0.201404	-3.308582
1	4.300430	-0.016069	-2.530375
1	3.009989	1.144102	-2.198349

(*R*,*P*)-**5b**	Coordinates (Angstroms)		
	X	Y	Z
O	-0.218616	-1.252188	1.537541
O	1.823930	-0.715450	2.021078
O	-3.544007	0.621569	-1.339854
H	-4.321899	0.335234	-1.828724
C	0.186345	1.251588	-0.027231
C	-1.356541	-1.324522	0.770285
C	-3.052512	-0.417042	-0.624734
C	0.959686	-0.680433	1.192687
C	-1.201216	1.108183	0.152745
C	-1.980773	2.242970	0.384821
H	-3.041644	2.123226	0.553679
C	0.723760	-0.859641	-1.375961
H	-0.334702	-1.075250	-1.461986
H	1.023940	-0.331382	-2.281368
C	1.103614	0.021002	-0.168604
C	0.723088	2.538643	-0.001299
H	1.783189	2.685853	-0.138171
C	2.587941	0.414853	-0.315470
H	2.670092	1.054753	-1.197728
H	2.871368	1.019827	0.544448
C	-1.877510	-0.210344	0.119353
C	-1.427768	3.507589	0.412552
H	-2.055396	4.369808	0.599601
C	-3.674922	-1.657548	-0.660485
H	-4.578883	-1.781478	-1.245419
C	-0.067453	3.655618	0.202368
H	0.386883	4.638602	0.210593
C	-1.968103	-2.566907	0.753361
H	-1.515607	-3.384429	1.297494
C	-3.136677	-2.725750	0.035067
H	-3.628242	-3.689617	0.006003

H	1.256026	-1.810221	-1.343510
C	3.598749	-0.746304	-0.452115
H	3.135042	-1.668582	-0.092494
C	4.822463	-0.484755	0.421750
H	5.549973	-1.295333	0.332550
H	5.320164	0.442881	0.122159
H	4.538986	-0.394041	1.471465
C	4.034762	-0.968094	-1.899710
H	3.192078	-1.175904	-2.560031
H	4.547457	-0.081091	-2.284041
H	4.729102	-1.808929	-1.970916

(*S*,*M*)-**5c**	Coordinates (Angstroms)		
	X	Y	Z
O	-0.093090	-1.364524	-1.441666
O	-3.298763	0.937484	1.254077
H	-4.118510	0.752204	1.722763
O	1.996602	-1.005804	-1.894002
C	-1.251283	-1.297275	-0.704511
C	-2.891944	-0.173665	0.596901
C	-1.531396	2.329279	-0.472318
H	-2.594292	2.310005	-0.668127
C	-1.680441	-0.112180	-0.114124
C	0.510943	1.144696	0.049216
C	1.121387	-0.882359	-1.085824
C	-3.633878	-1.345577	0.659472
H	-4.563536	-1.357312	1.217118
C	-0.877771	1.132684	-0.172755
C	0.779000	-0.977018	1.475739
H	1.247913	-0.571990	2.373456
H	-0.293849	-0.931968	1.623035
C	-3.180590	-2.489039	0.025405
H	-3.764025	-3.399390	0.074728
C	0.511737	3.548148	-0.288543
H	1.064186	4.478895	-0.324128
C	1.290130	-0.166369	0.264244
C	-0.851050	3.529261	-0.532310
H	-1.382348	4.441887	-0.771545
C	2.797997	0.075584	0.470729
H	3.210202	0.577430	-0.405142
H	2.907568	0.758719	1.317439
C	-1.981053	-2.473445	-0.658394
H	-1.595113	-3.352895	-1.155304

C	1.178518	2.367117	-0.014937
H	2.244814	2.410532	0.145913
C	3.635981	-1.170154	0.751705
H	3.356727	-1.601762	1.715360
H	3.441155	-1.931877	-0.005993
C	5.123052	-0.843141	0.765797
H	5.351702	-0.078714	1.513563
H	5.719792	-1.726941	1.000279
H	5.450669	-0.466751	-0.206406
H	1.062933	-2.026604	1.392767

(*S*,*P*)-**5c**	Coordinates (Angstroms)		
	X	Y	Z
O	-0.575714	-0.627318	1.806673
O	0.383410	-2.564959	1.996725
O	-0.269864	3.078213	-1.192707
H	-0.839292	3.754243	-1.573277
C	1.581390	-0.328692	-0.189155
C	-1.111445	0.435113	1.114543
C	-0.985543	2.313324	-0.335683
C	0.137900	-1.646484	1.269503
C	1.108394	0.967975	0.077249
C	2.014151	2.027555	0.143260
H	1.645318	3.017742	0.371843
C	-0.514541	-1.413856	-1.215763
H	-0.832594	-0.381041	-1.330089
H	-0.089243	-1.705200	-2.179644
C	0.639451	-1.543799	-0.180280
C	2.947999	-0.498284	-0.406610
H	3.344061	-1.480732	-0.614675
C	1.372843	-2.853668	-0.482374
H	1.801807	-2.813263	-1.483983
H	0.672565	-3.686793	-0.448571
H	2.162860	-3.065816	0.234985
C	-0.327733	1.248947	0.304077
C	3.365119	1.836531	-0.070237
H	4.047522	2.674989	-0.009060
C	-2.337345	2.552567	-0.129153
H	-2.813769	3.381069	-0.640750
C	-1.743934	-2.271218	-0.929765
H	-1.468764	-3.327635	-0.883529
H	-2.156919	-2.015859	0.051064
C	3.831773	0.565608	-0.359775

H	4.886056	0.394295	-0.538565
C	-2.459051	0.662258	1.338486
H	-3.004094	-0.002409	1.994554
C	-3.068301	1.731731	0.711801
H	-4.121478	1.924445	0.870606
C	-2.823277	-2.070119	-1.983502
H	-3.697692	-2.689986	-1.776540
H	-2.455752	-2.331112	-2.979233
H	-3.150349	-1.027358	-2.010401

(*R*,*M*)-**5d**	Coordinates (Angstroms)		
	X	Y	Z
O	-0.236742	-1.317394	-1.467692
O	-2.060839	2.325933	1.029934
H	-2.897789	2.585070	1.428249
O	1.672020	-2.343576	-1.573939
C	-1.266598	-0.661343	-0.832268
C	0.968886	-1.620823	-0.928876
C	-1.085111	0.579946	-0.232233
C	-2.218026	1.155296	0.368640
C	-2.495089	-1.297460	-0.898633
H	-2.567739	-2.253637	-1.398376
C	1.411289	0.525566	0.151187
C	-3.592730	-0.690951	-0.320153
H	-4.560930	-1.173263	-0.358222
C	0.243081	1.232616	-0.181935
C	0.329222	2.590710	-0.491717
H	-0.569694	3.121520	-0.772434
C	0.447652	-1.384362	1.586663
H	1.016512	-1.188338	2.497948
H	-0.424781	-0.739545	1.632895
C	-3.455674	0.528404	0.319706
H	-4.308351	1.000219	0.794468
C	1.387463	-0.989619	0.409052
C	2.776039	-1.546053	0.737563
H	2.716900	-2.621160	0.899207
H	3.150120	-1.087297	1.653227
H	3.493563	-1.379070	-0.063041
C	1.533849	3.264825	-0.455145
H	1.573540	4.317845	-0.704075
C	2.616004	1.226813	0.187055
H	3.531729	0.714602	0.440593
C	2.682767	2.578788	-0.100122

H	3.636970	3.089097	-0.056935
C	-0.011060	-2.836996	1.574665
H	-0.651734	-3.052027	0.715854
H	-0.592456	-3.050169	2.473115
H	0.826843	-3.535691	1.550414

(*R*,*P*)-**5d**	Coordinates (Angstroms)		
	X	Y	Z
O	-0.048824	-1.470927	-1.349149
O	-2.191997	-1.476967	-1.676766
O	2.879733	1.426061	1.075135
H	3.752232	1.401509	1.480439
C	-0.978357	0.950956	0.121586
C	1.122714	-1.185857	-0.689079
C	2.626281	0.240605	0.473483
C	-1.303761	-1.184520	-0.928642
C	0.376522	1.164447	-0.189344
C	0.799275	2.443611	-0.555099
H	1.836596	2.596457	-0.817941
C	-0.798559	-1.139561	1.606520
H	0.256193	-0.903433	1.687834
H	-1.280320	-0.797376	2.523419
C	-1.510488	-0.464274	0.413428
C	-1.845574	2.041686	0.075590
H	-2.891991	1.910327	0.304763
C	-3.023878	-0.472680	0.711173
H	-3.193402	0.207306	1.549239
H	-3.565005	-0.073618	-0.145891
C	1.380078	0.073909	-0.155966
C	-0.076669	3.510069	-0.596509
H	0.277160	4.490728	-0.888954
C	3.558215	-0.787530	0.514652
H	4.508855	-0.622905	1.008936
C	-1.405870	3.308389	-0.265409
H	-2.108585	4.132186	-0.284896
C	2.042258	-2.221162	-0.662760
H	1.781242	-3.169391	-1.112219
C	3.266723	-2.012303	-0.059578
H	3.997395	-2.810234	-0.026459
H	-0.897366	-2.224536	1.559994
C	-3.606777	-1.835686	1.067571
H	-4.690868	-1.752193	1.163657

H	-3.401587	-2.579507	0.296929
H	-3.224627	-2.211246	2.017706

(*R*,*M*)-**5f**	Coordinates (Angstroms)		
	X	Y	Z
O	0.819591	0.973280	-1.410548
O	-0.735810	2.442011	-1.765857
C	1.535610	0.048914	-0.684694
C	-0.304621	1.614583	-1.015455
C	0.946232	-1.050225	-0.070288
C	1.799958	-1.951169	0.572220
C	2.908619	0.240612	-0.682127
H	3.313451	1.106396	-1.189945
C	-1.435512	-0.222837	0.152985
C	3.729450	-0.668625	-0.042367
H	4.800948	-0.515201	-0.035735
C	-0.516509	-1.266529	-0.051912
C	-0.985100	-2.570413	-0.221450
H	-0.271917	-3.363686	-0.406977
C	-0.027896	1.420769	1.537070
H	-0.679524	1.459866	2.412464
H	0.613720	0.555864	1.679044
C	3.170050	-1.770384	0.590382
H	3.800557	-2.484916	1.103803
C	-0.974317	1.234407	0.313933
C	-2.149912	2.197849	0.500722
H	-1.782965	3.218552	0.594389
H	-2.690604	1.949369	1.414268
H	-2.841721	2.178860	-0.338901
C	-2.333986	-2.865253	-0.180717
H	-2.671904	-3.884208	-0.321461
C	-2.791958	-0.542260	0.187590
H	-3.525877	0.234016	0.342305
C	-3.241930	-1.842153	0.031427
H	-4.304274	-2.048752	0.068172
C	0.837386	2.673565	1.487783
H	1.566958	2.629257	0.675322
H	1.396407	2.775018	2.419299
H	0.246060	3.581388	1.357150
H	1.367042	-2.799270	1.087422

(*R*,*P*)-**5f**	Coordinates (Angstroms)		
	X	Y	Z
O	-0.460233	-1.379117	1.257867
O	1.619479	-1.691020	1.786418
C	0.994199	0.859813	-0.146410
C	-1.510639	-0.857997	0.540707
C	-2.667195	0.848617	-0.642122
C	0.856112	-1.281638	0.959229
C	-0.320232	1.316980	0.056724
C	-0.541966	2.674168	0.298605
H	-1.552809	3.015312	0.482835
C	0.556044	-1.204876	-1.600378
H	-0.437581	-0.799916	-1.756049
H	1.147868	-0.963931	-2.484510
C	1.288134	-0.635277	-0.365247
C	2.029589	1.791652	-0.098405
H	3.050733	1.472596	-0.240958
C	2.794070	-0.914899	-0.545697
H	3.139589	-0.303427	-1.382378
H	3.333149	-0.585699	0.341926
C	-1.500965	0.425326	0.002534
C	0.497743	3.582734	0.333566
H	0.298319	4.628620	0.530043
C	-3.786285	0.042960	-0.732408
H	-4.668634	0.402546	-1.246004
C	1.791908	3.136353	0.128092
H	2.623135	3.829942	0.152964
C	-2.627987	-1.675465	0.465590
H	-2.582989	-2.658831	0.915406
C	-3.768468	-1.227165	-0.172238
H	-4.638162	-1.868678	-0.234736
H	0.469510	-2.290134	-1.538916
C	3.154565	-2.368727	-0.829589
H	4.240658	-2.476041	-0.846472
H	2.770113	-3.040778	-0.061656
H	2.780231	-2.703813	-1.797802
H	-2.678849	1.827960	-1.103290

TS-**5b**	Coordinates (Angstroms)		
	X	Y	Z
C	3.021206	-0.537783	-0.124302
C	3.543217	-1.809762	-0.310189
C	2.731936	-2.922493	-0.223435
C	1.419423	-2.751825	0.143837
C	0.909701	-1.472299	0.336116
C	1.625944	-0.290608	0.054053
C	1.044391	1.095105	-0.092219
C	-0.267323	1.522335	0.246490
C	-0.587583	2.876258	0.138811
C	0.276254	3.817669	-0.381927
C	1.495924	3.387117	-0.860954
C	1.854830	2.063367	-0.712250
O	-0.328586	-1.532506	0.909181
C	-1.445053	0.585059	0.542445
C	-0.978619	-0.490458	1.491432
O	-1.214870	-0.548706	2.659869
H	4.605443	-1.914105	-0.500916
H	3.137298	-3.913687	-0.379072
H	0.767952	-3.590910	0.344802
H	-1.567361	3.209688	0.441662
H	-0.023591	4.855957	-0.447512
H	2.180760	4.071910	-1.345326
H	2.812032	1.776172	-1.102438
O	3.903549	0.491036	-0.076529
H	4.797097	0.137157	-0.125689
C	-1.870071	-0.041845	-0.811786
H	-2.197527	0.784965	-1.447782
C	-2.640244	1.273889	1.208918
H	-3.362659	0.522462	1.525221
H	-2.342189	1.836935	2.092348
H	-0.984807	-0.456953	-1.295036
C	-2.970594	-1.121046	-0.746218
H	-3.022744	-1.512781	0.275545
C	-2.622300	-2.289123	-1.664188
H	-1.671321	-2.743812	-1.378250
H	-3.393187	-3.062489	-1.627962
H	-2.535291	-1.951672	-2.701368
C	-4.346711	-0.561511	-1.099426

H	-4.358871	-0.204780	-2.133561
H	-5.116555	-1.330740	-1.002944
H	-4.626579	0.273625	-0.456020
H	-3.144605	1.942194	0.512895

TS-**5c**	Coordinates (Angstroms)		
	X	Y	Z
C	-2.785419	0.697599	-0.159588
C	-3.861422	-0.073278	-0.575007
C	-3.726272	-1.436563	-0.741266
C	-2.534145	-2.027009	-0.399077
C	-1.465293	-1.245521	0.025981
C	-1.472727	0.164044	0.016649
C	-0.275215	1.070205	0.158895
C	1.026636	0.719492	0.603072
C	1.954773	1.733349	0.840683
C	1.716213	3.058773	0.538387
C	0.524426	3.381808	-0.077289
C	-0.435182	2.405474	-0.251469
O	-0.459484	-2.031359	0.513706
C	1.571468	-0.712091	0.644268
C	0.551159	-1.599922	1.315138
O	0.613197	-2.024531	2.428818
H	-4.816584	0.411113	-0.743219
H	-4.562861	-2.036126	-1.075544
H	-2.406865	-3.100598	-0.399152
H	2.924452	1.474598	1.235787
H	2.472807	3.807893	0.734718
H	0.323602	4.391252	-0.413778
H	-1.350286	2.703211	-0.727328
O	-3.027223	2.002102	0.121617
H	-3.970420	2.171030	0.035028
C	2.872394	-0.872658	1.436212
H	3.700866	-0.351615	0.963085
H	3.133030	-1.930775	1.478383
C	1.774680	-1.202414	-0.818842
H	2.039865	-2.264162	-0.784123
H	0.824992	-1.136504	-1.352395
H	2.769580	-0.510410	2.458020
C	2.823974	-0.449026	-1.627515
H	2.596824	0.619853	-1.624336

H	3.810324	-0.562902	-1.172590
C	2.877642	-0.949002	-3.065159
H	1.917182	-0.806512	-3.566732
H	3.636797	-0.414088	-3.639150
H	3.118632	-2.014521	-3.104232

TS-**5d**	Coordinates (Angstroms)		
	X	Y	Z
O	-0.460233	-1.379117	1.257867
O	1.619479	-1.691020	1.786418
C	0.994199	0.859813	-0.146410
C	-1.510639	-0.857997	0.540707
C	-2.667195	0.848617	-0.642122
C	0.856112	-1.281638	0.959229
C	-0.320232	1.316980	0.056724
C	-0.541966	2.674168	0.298605
H	-1.552809	3.015312	0.482835
C	0.556044	-1.204876	-1.600378
H	-0.437581	-0.799916	-1.756049
H	1.147868	-0.963931	-2.484510
C	1.288134	-0.635277	-0.365247
C	2.029589	1.791652	-0.098405
H	3.050733	1.472596	-0.240958
C	2.794070	-0.914899	-0.545697
H	3.139589	-0.303427	-1.382378
H	3.333149	-0.585699	0.341926
C	-1.500965	0.425326	0.002534
C	0.497743	3.582734	0.333566
H	0.298319	4.628620	0.530043
C	-3.786285	0.042960	-0.732408
H	-4.668634	0.402546	-1.246004
C	1.791908	3.136353	0.128092
H	2.623135	3.829942	0.152964
C	-2.627987	-1.675465	0.465590
H	-2.582989	-2.658831	0.915406
C	-3.768468	-1.227165	-0.172238
H	-4.638162	-1.868678	-0.234736
H	0.469510	-2.290134	-1.538916
C	3.154565	-2.368727	-0.829589
H	4.240658	-2.476041	-0.846472

H	2.770113	-3.040778	-0.061656
H	2.780231	-2.703813	-1.797802
H	-2.678849	1.827960	-1.103290

TS-**5f**	Coordinates (Angstroms)		
	X	Y	Z
C	-2.494684	1.468450	-0.045370
C	-3.805973	1.083116	0.134661
C	-4.110354	-0.251452	0.355578
C	-3.085696	-1.168515	0.327424
C	-1.770211	-0.764341	0.121854
C	-1.399791	0.581971	-0.004429
C	0.001578	1.145638	-0.097971
C	1.218666	0.437626	-0.266855
C	2.398766	1.161870	-0.448456
C	2.454911	2.539349	-0.408301
C	1.291790	3.236647	-0.150400
C	0.109209	2.543728	-0.001499
O	-0.900706	-1.819131	0.053911
C	1.393298	-1.084611	-0.138730
C	0.188040	-1.741608	-0.769442
O	0.136422	-2.225285	-1.857667
H	-4.588737	1.829532	0.094940
H	-5.131271	-0.576161	0.510187
H	-3.272724	-2.228681	0.436730
H	3.322339	0.626499	-0.604071
H	3.398892	3.050342	-0.548948
H	1.293447	4.315904	-0.064328
H	-0.762769	3.136384	0.216806
C	1.447590	-1.462047	1.369685
H	1.440812	-2.553776	1.439749
H	0.534093	-1.111828	1.849834
C	2.641646	-0.913217	2.136285
H	2.668981	0.177447	2.115540
H	2.570287	-1.220863	3.181081
H	3.592627	-1.285203	1.752283
C	2.620842	-1.636192	-0.871854
H	3.551008	-1.325082	-0.403532
H	2.591883	-2.726143	-0.842587
H	2.637085	-1.326150	-1.915656
H	-2.329441	2.514642	-0.241049

NMR Spectra and HRMS spectra

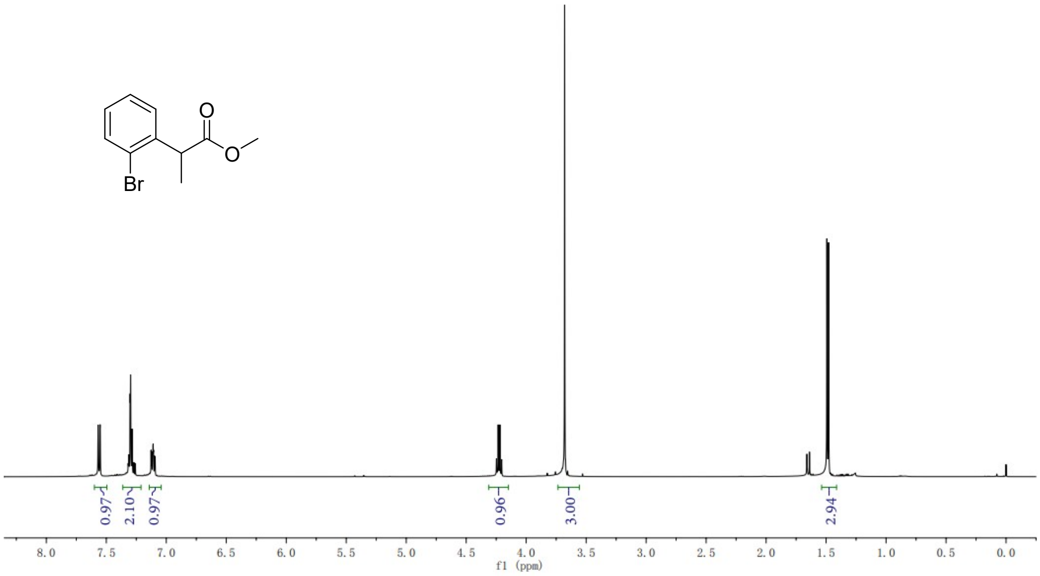

Figure S3. ^{1}H NMR (500 MHz, $CDCl_3$) spectrum of **3**.

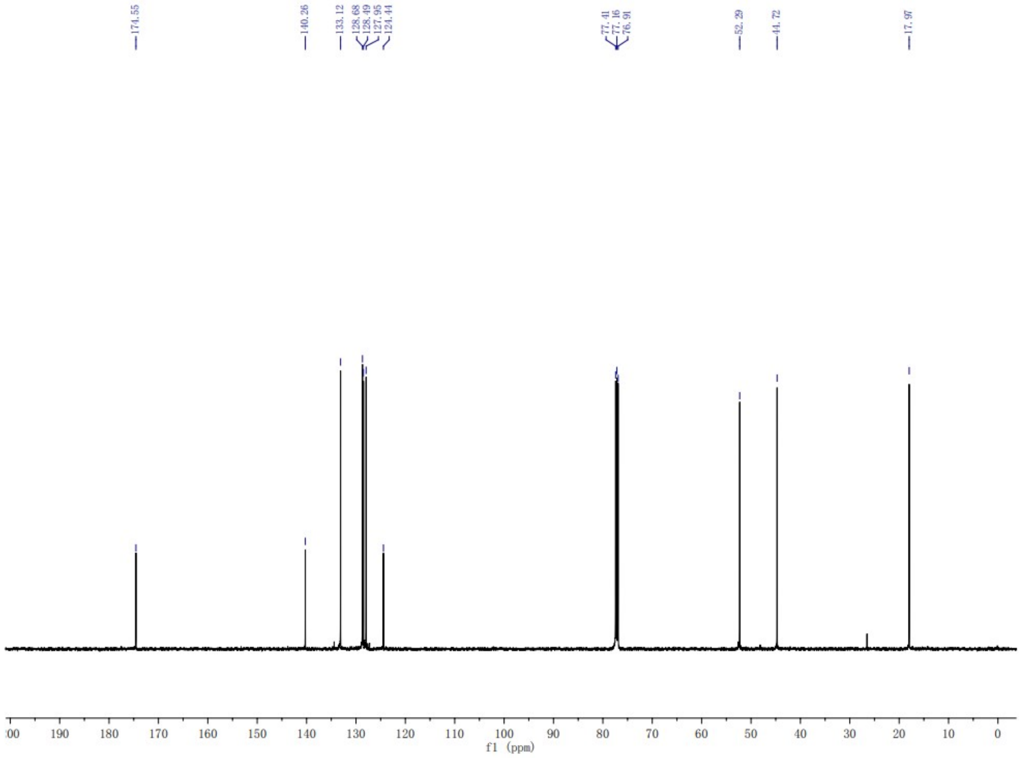

Figure S4. ^{13}C NMR (126 MHz, $CDCl_3$) spectrum of **3**.

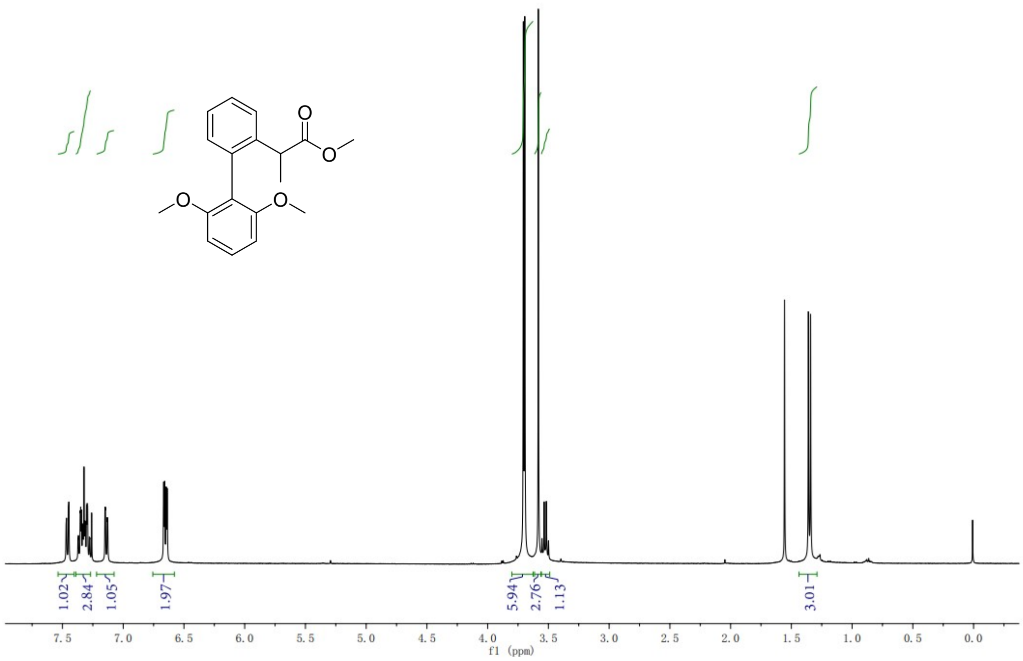

Figure S5. ^{1}H NMR (500 MHz, $CDCl_3$) spectrum of **4a**.

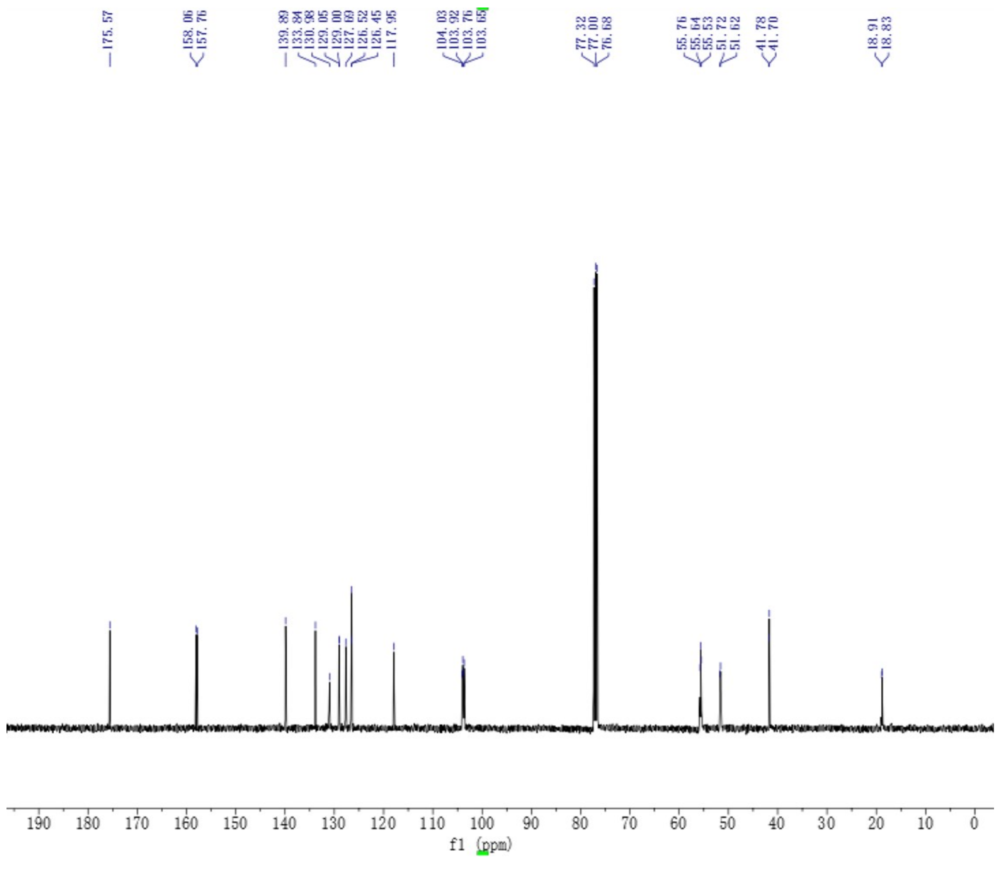

Figure S6. ^{13}C NMR (126 MHz, $CDCl_3$) spectrum of **4a**.

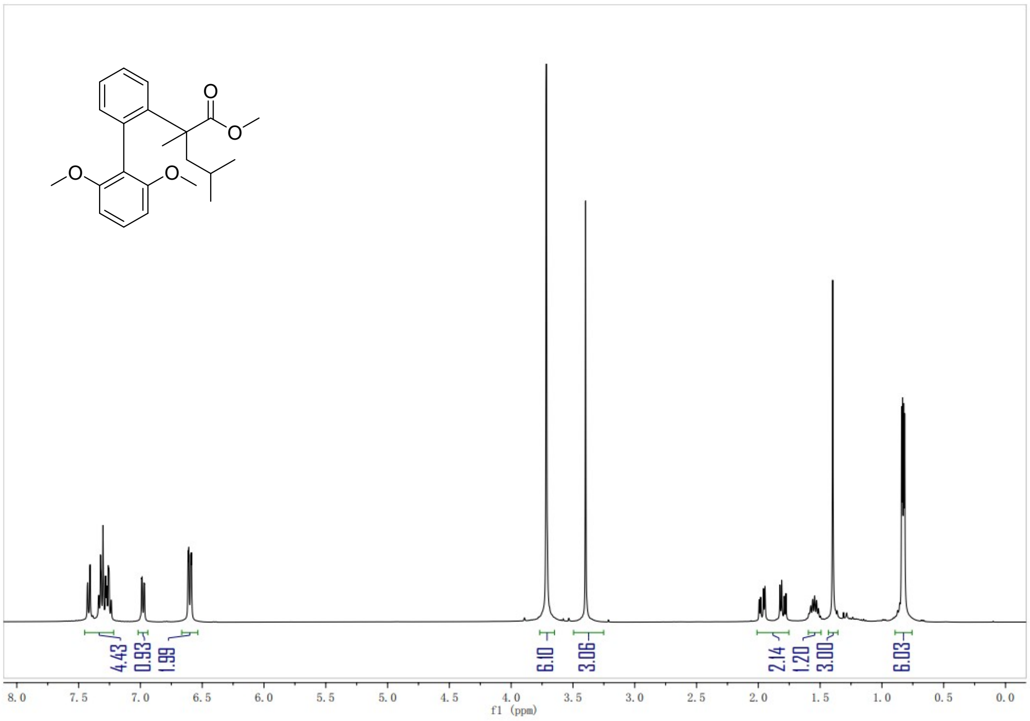

Figure S7. ^{1}H NMR (400 MHz, $CDCl_3$) spectrum of **4b**.

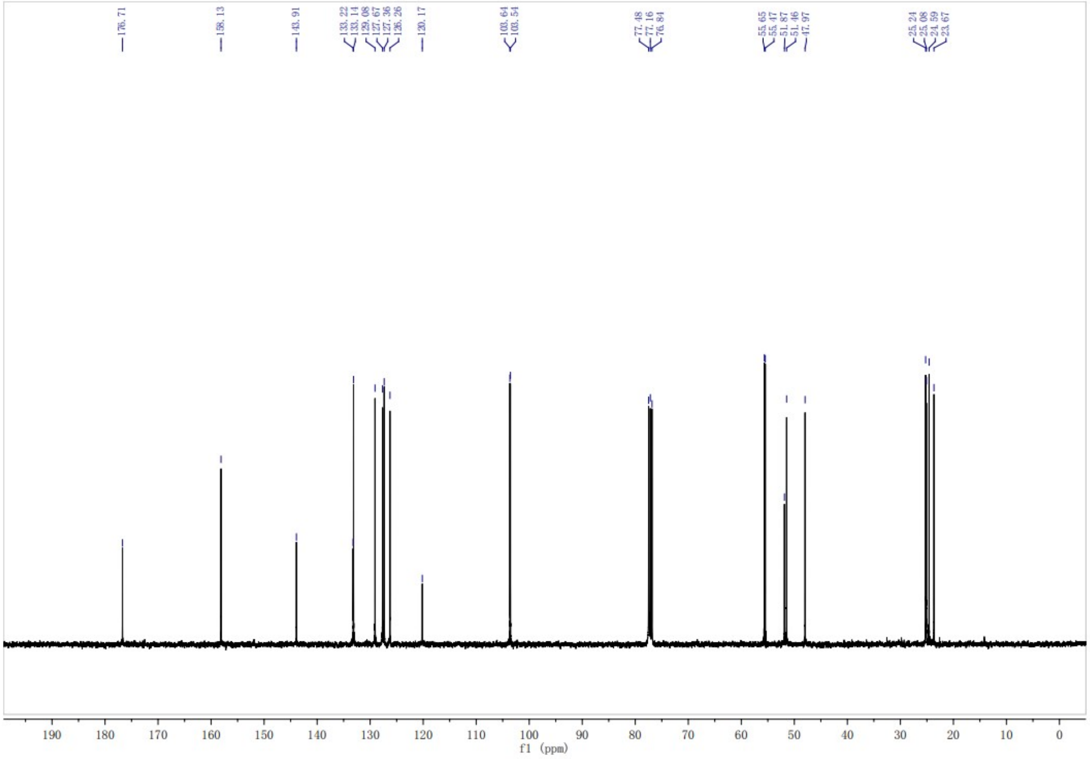

Figure S8. ^{13}C NMR (101 MHz, $CDCl_3$) spectrum of **4b**.

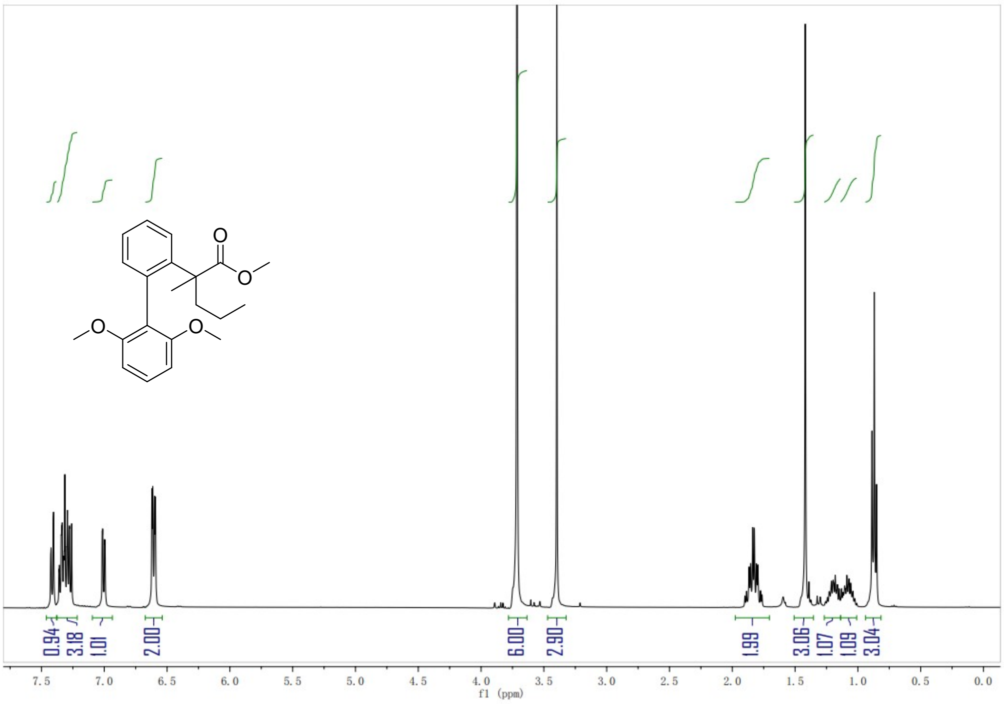

Figure S9. ^{1}H NMR (400 MHz, $CDCl_3$) spectrum of **4c**.

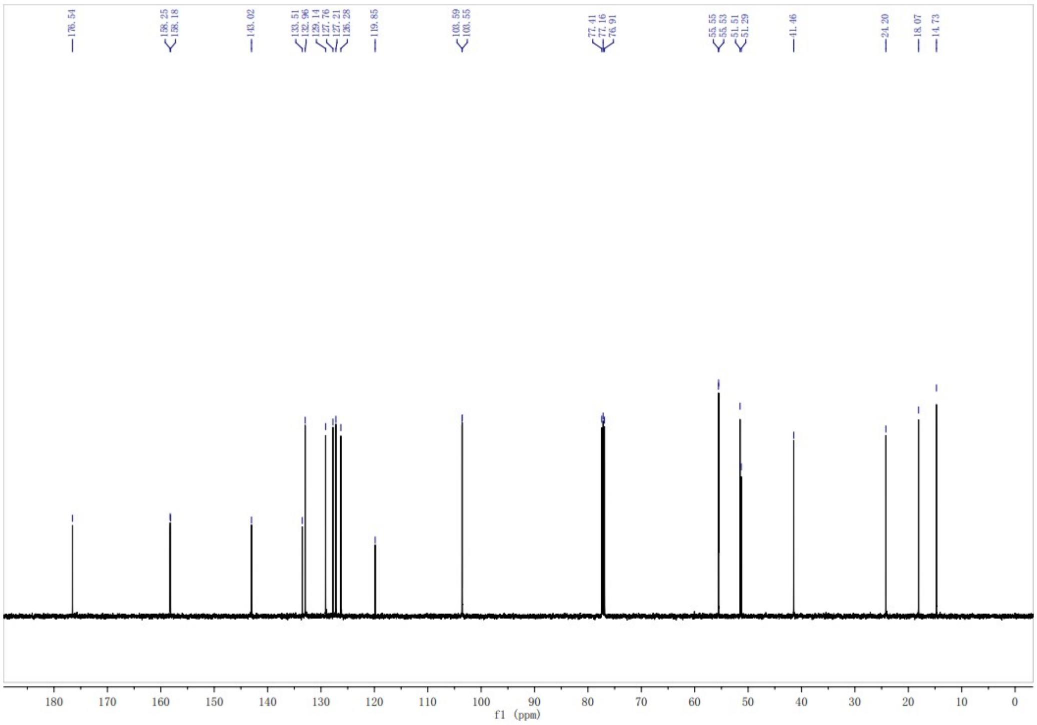

Figure S10. ^{13}C NMR (101 MHz, $CDCl_3$) spectrum of **4c**.

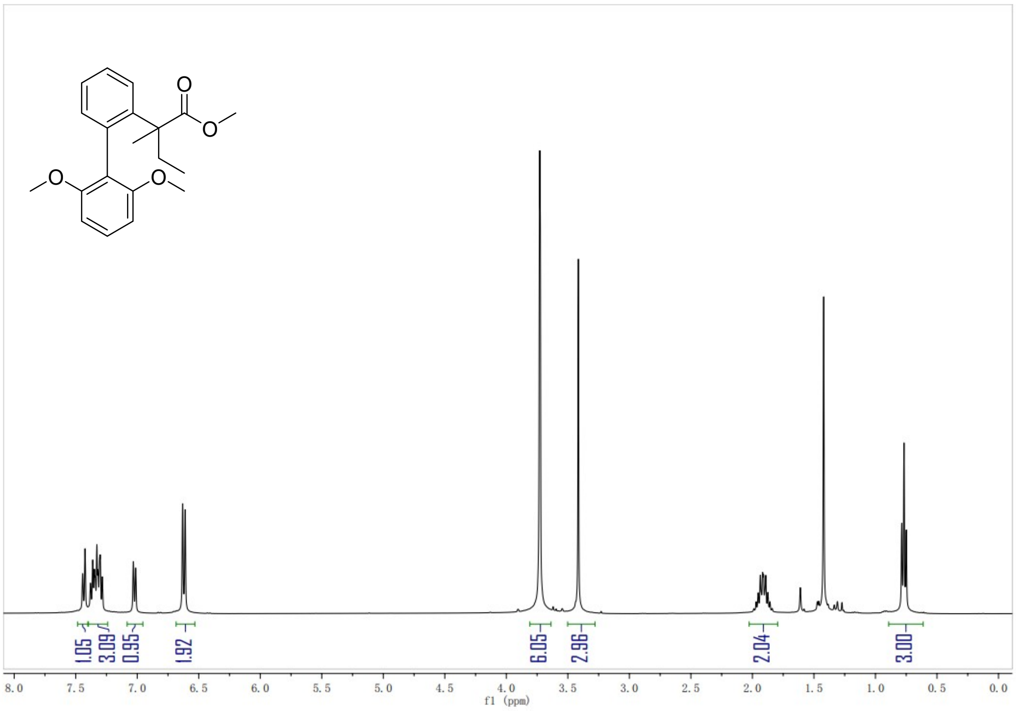

Figure S11. ^{1}H NMR (400 MHz, $CDCl_3$) spectrum of **4d**.

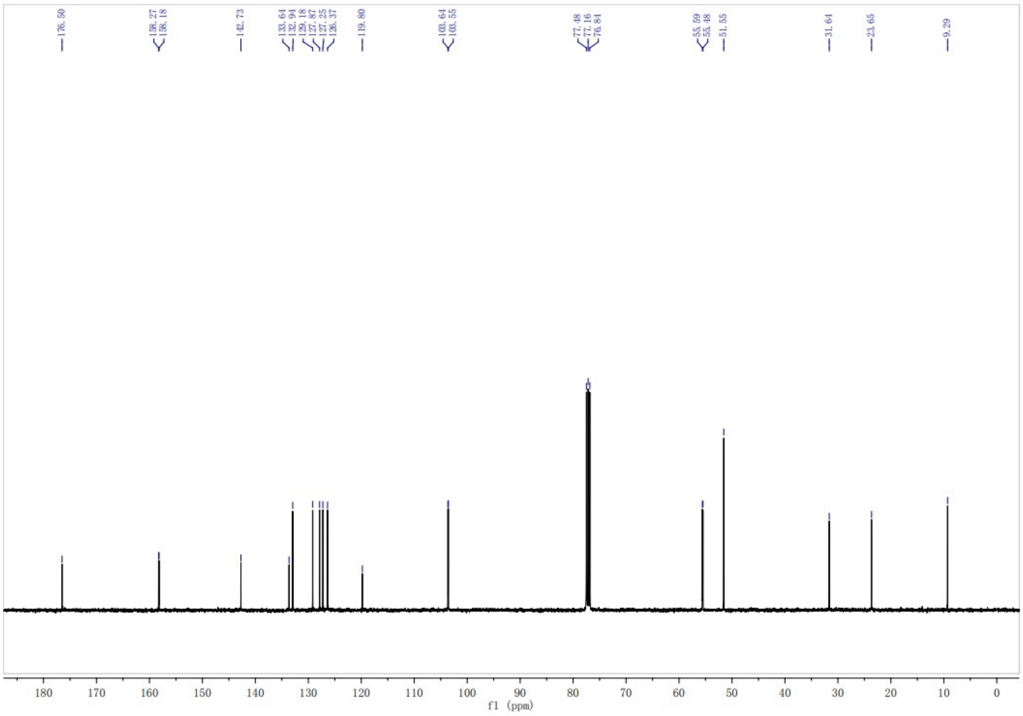

Figure S12. ^{13}C NMR (101 MHz, $CDCl_3$) spectrum of **4d**.

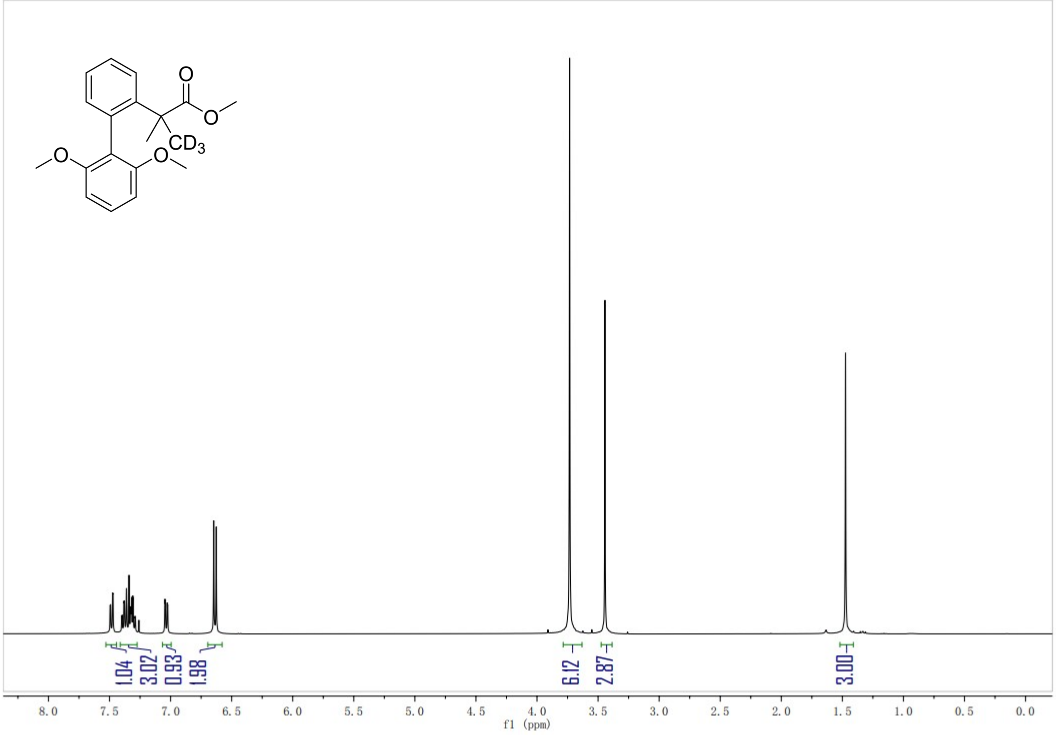

Figure S13. ^{1}H NMR (400 MHz, $CDCl_3$) spectrum of **4e**.

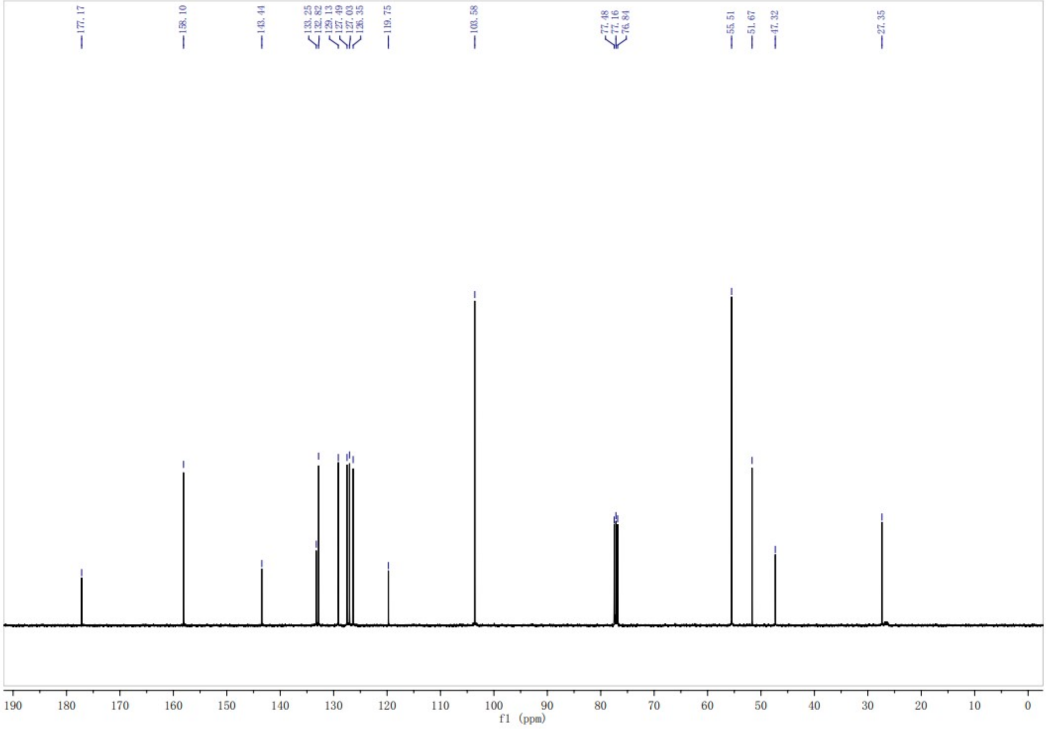

Figure S14. ^{13}C NMR (101 MHz, $CDCl_3$) spectrum of **4e**.

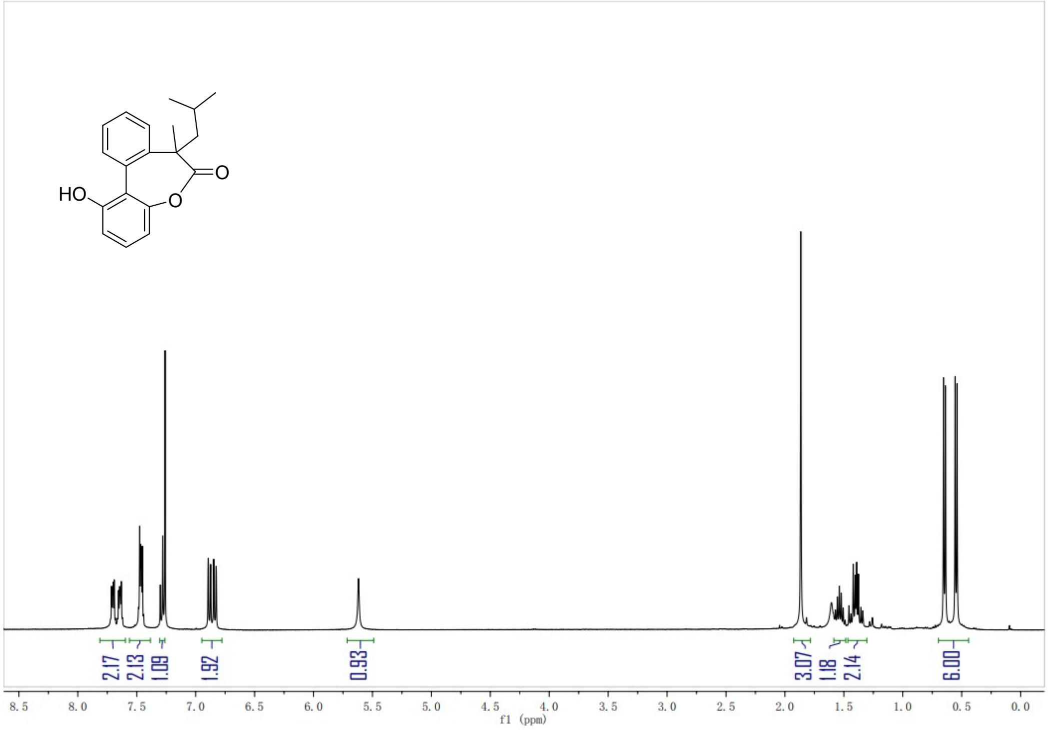

Figure S15. ^{1}H NMR (400 MHz, $CDCl_3$) spectrum of **5b**.

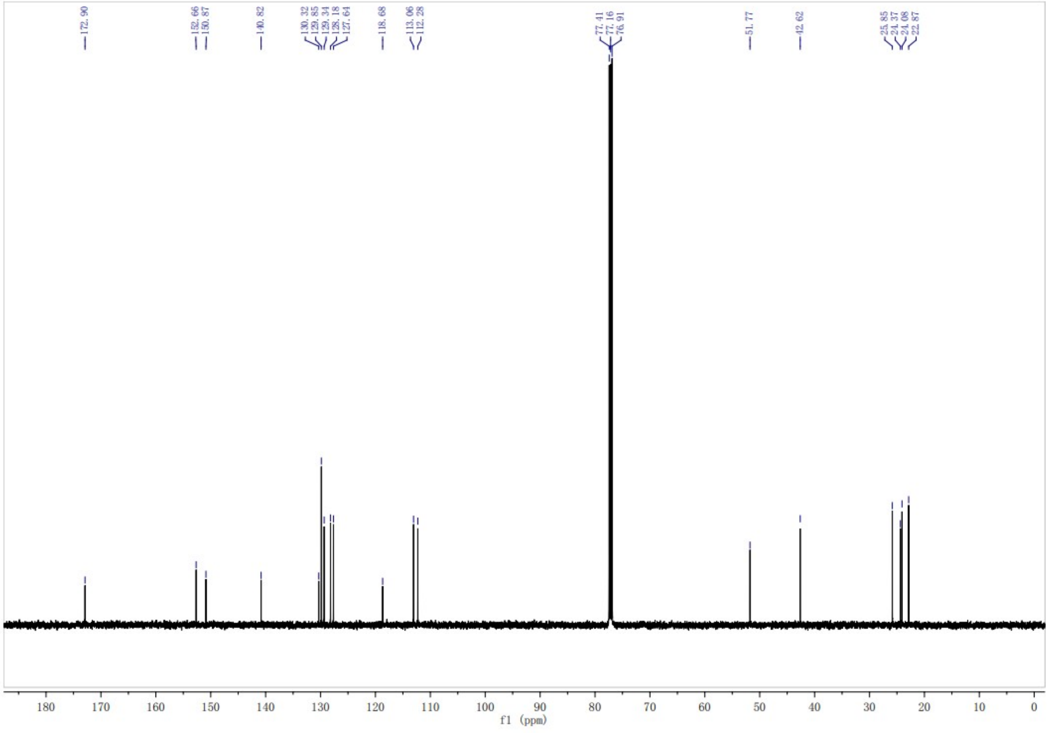

Figure S16. ^{13}C NMR (101 MHz, $CDCl_3$) spectrum of **5b**.

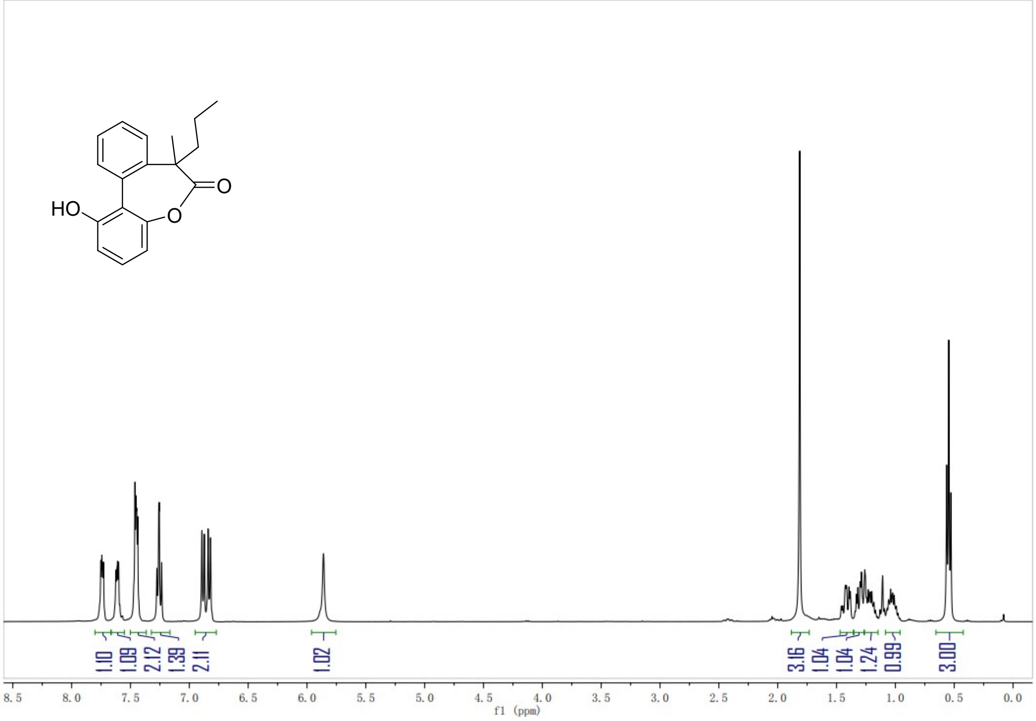

Figure S17. ^{1}H NMR (400 MHz, $CDCl_3$) spectrum of **5c**.

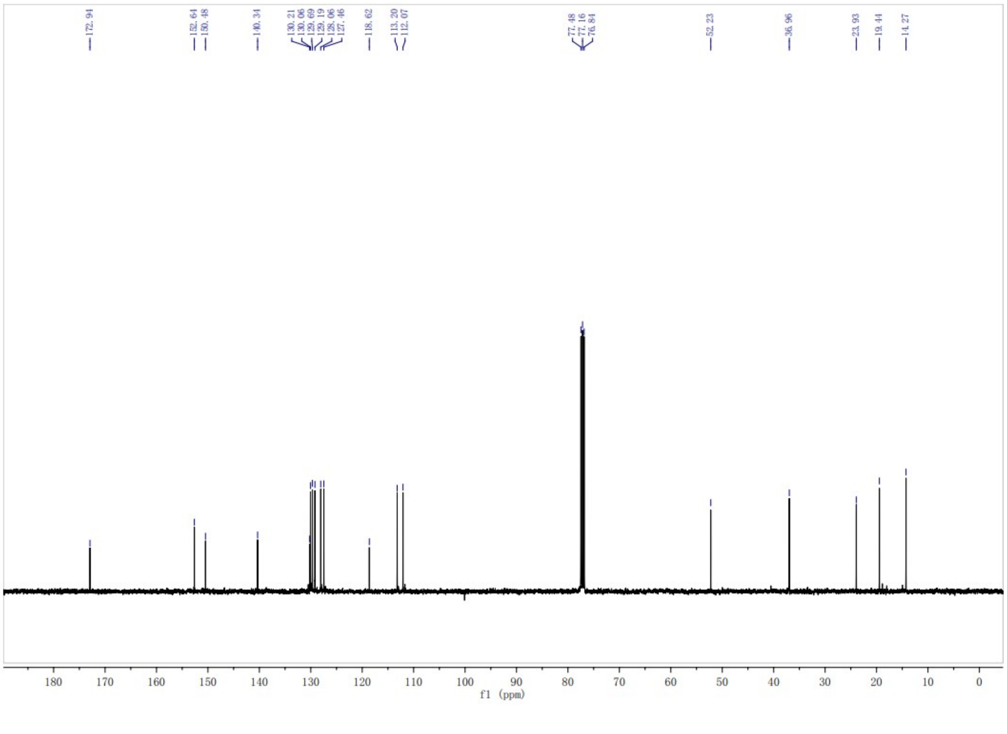

Figure S18. ^{13}C NMR (101 MHz, $CDCl_3$) spectrum of **5c**.

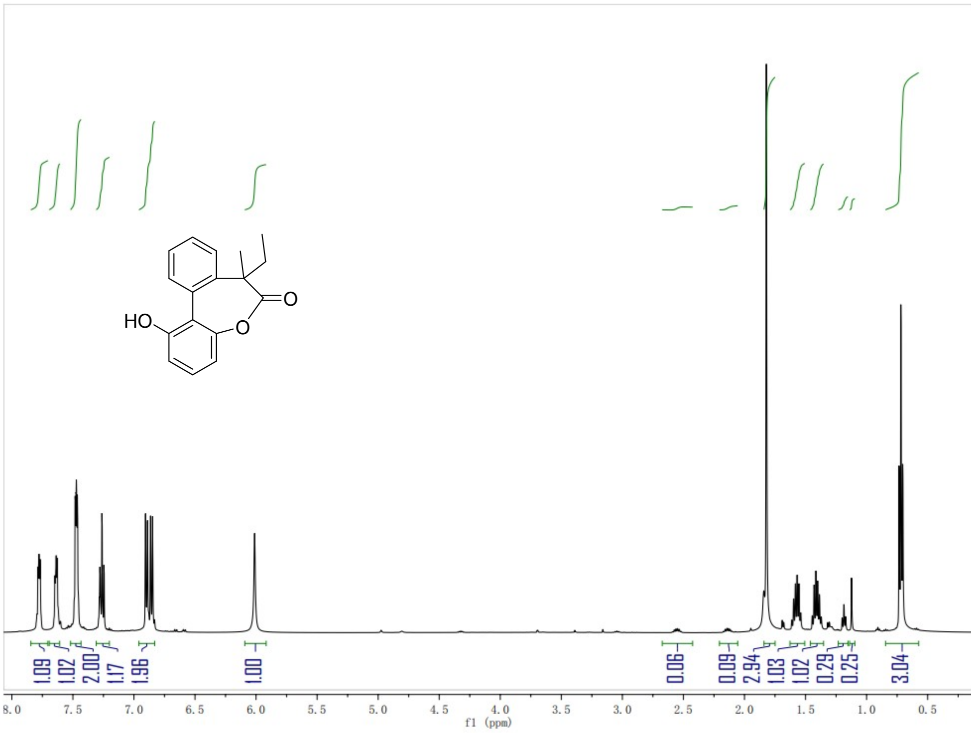

Figure S19. ^{1}H NMR (500 MHz, $CDCl_3$) spectrum of **5d**.

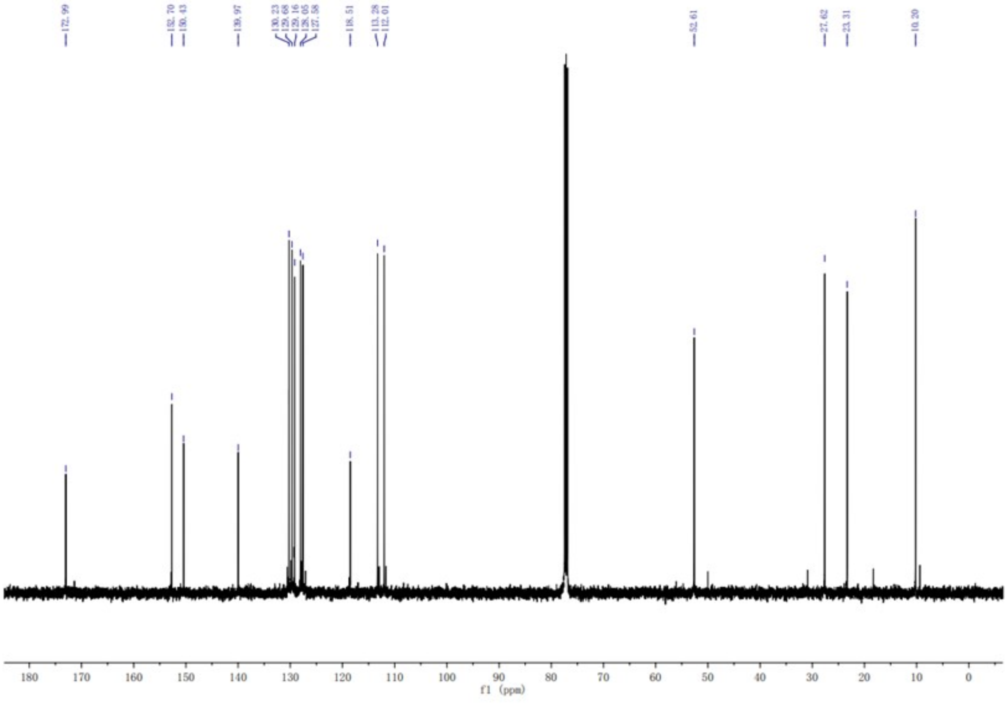

Figure S20. ^{13}C NMR (126 MHz, $CDCl_3$) spectrum of **5d**.

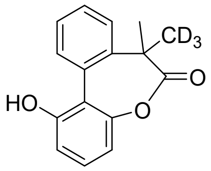

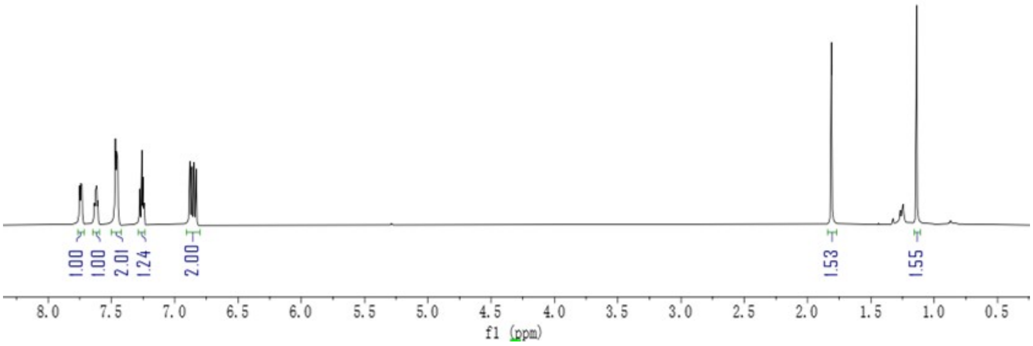

Figure S21. ^{1}H NMR (500 MHz, $CDCl_3$) spectrum of **5e**.

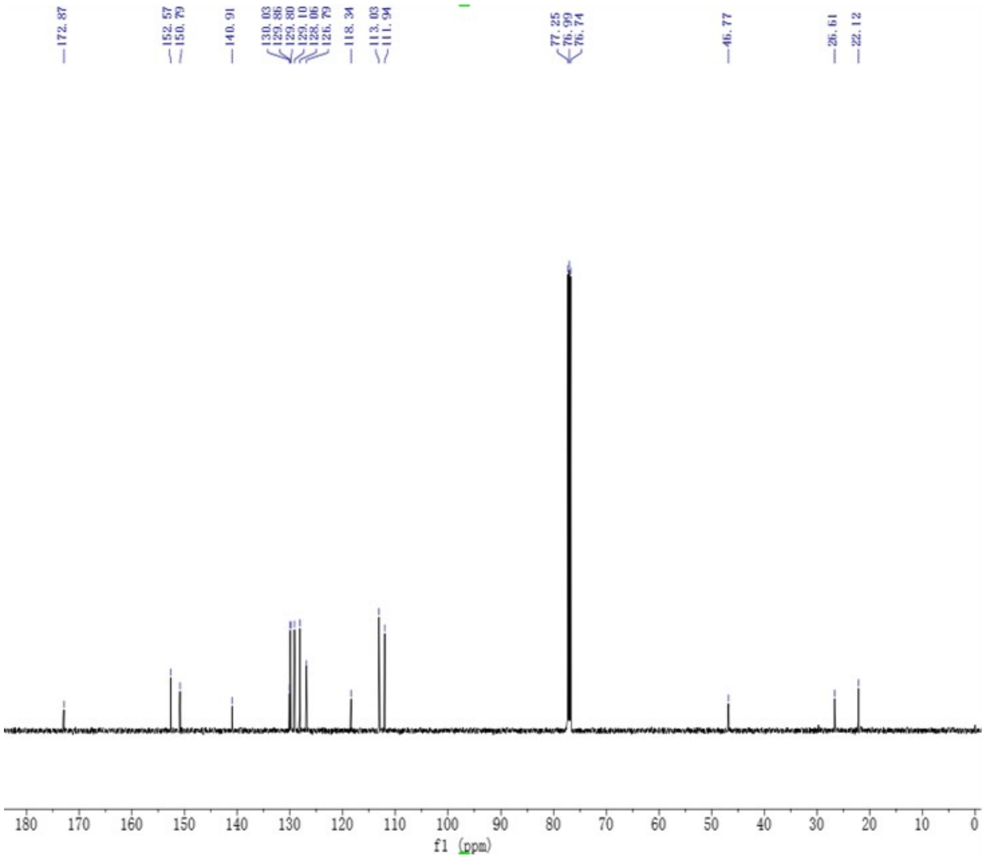

Figure S22. ^{13}C NMR (126 MHz, $CDCl_3$) spectrum of **5e**.

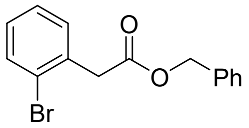

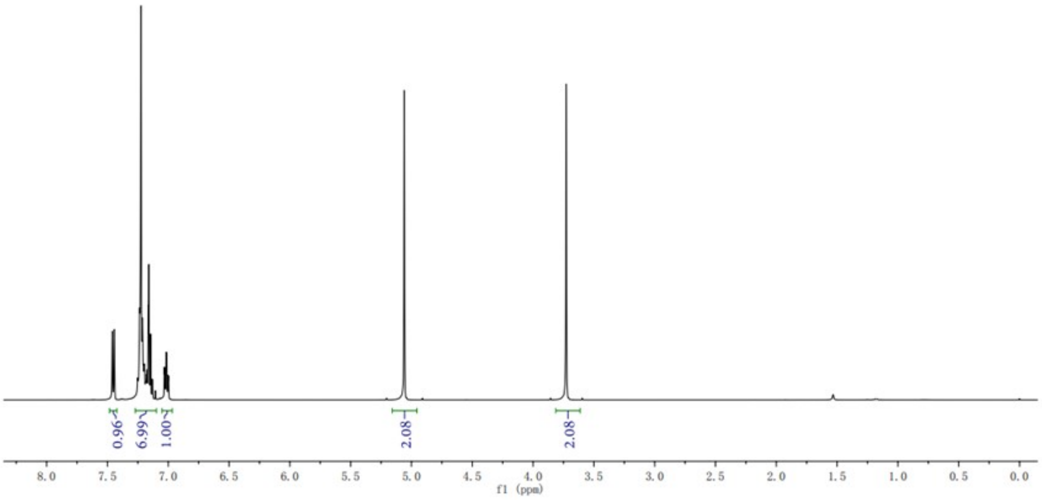

Figure S23. ^{1}H NMR (500 MHz, $CDCl_3$) spectrum of **6**.

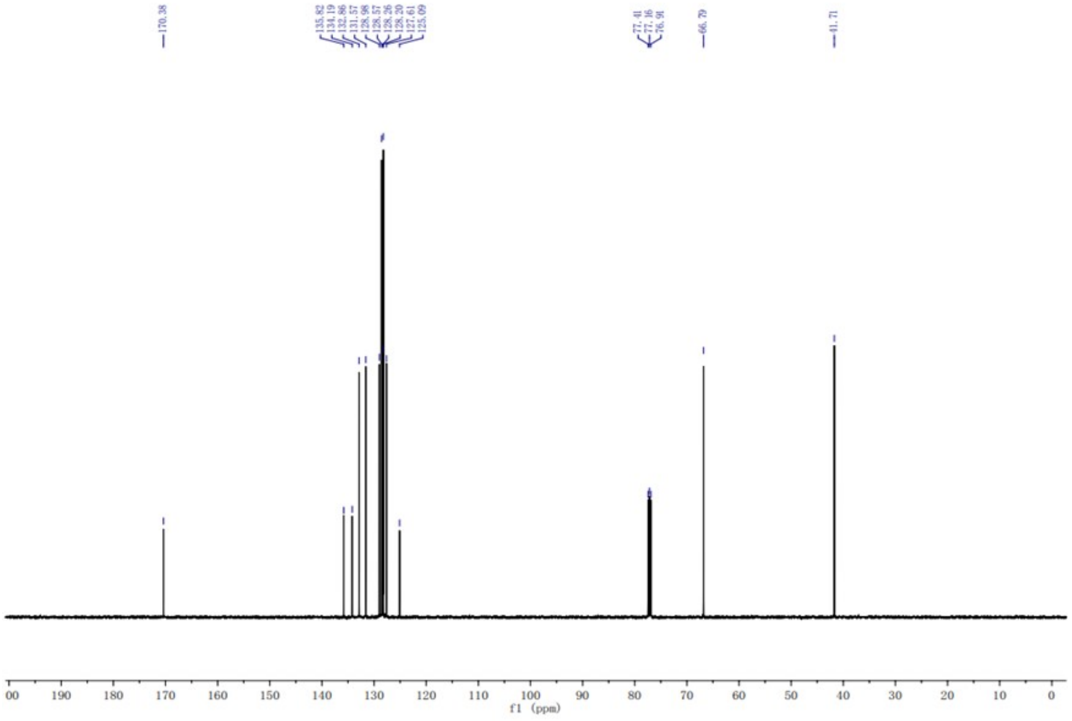

Figure S24. ^{13}C NMR (126 MHz, $CDCl_3$) spectrum of **6**.

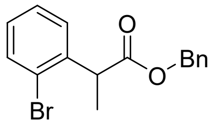

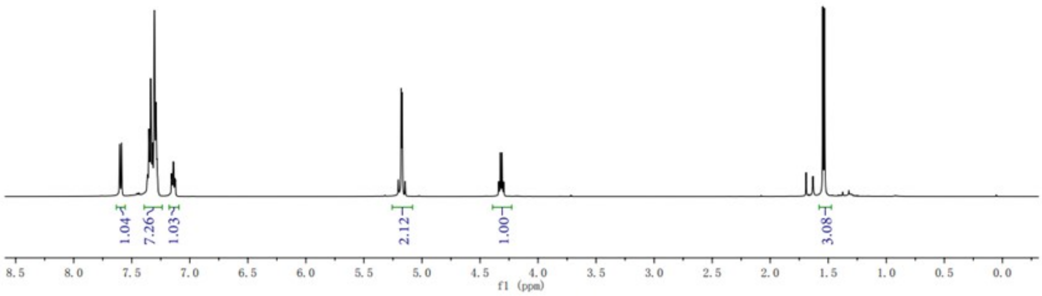

Figure S25. ^{1}H NMR (500 MHz, $CDCl_3$) spectrum of **7**.

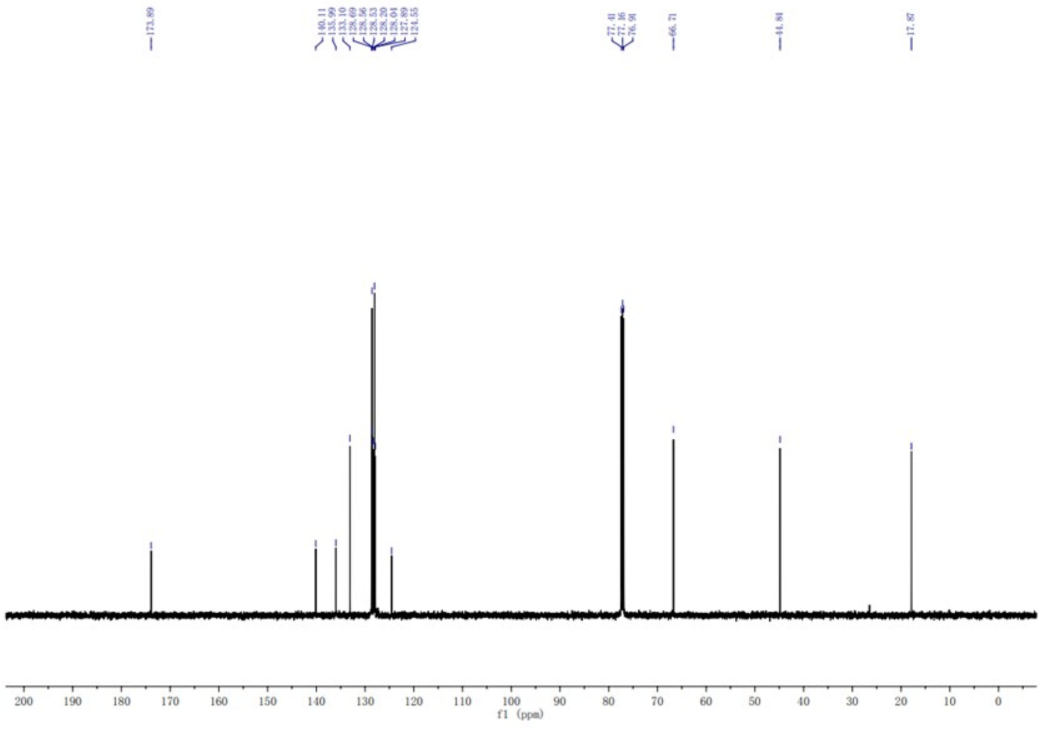

Figure S26. ^{13}C NMR (126 MHz, $CDCl_3$) spectrum of **7**.

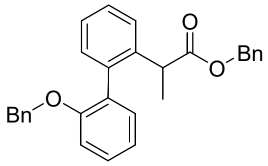

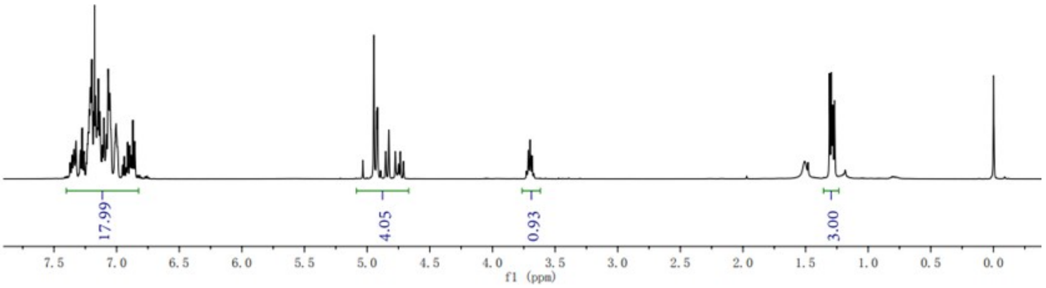

Figure S27. ^{1}H NMR (500 MHz, $CDCl_3$) spectrum of **8**.

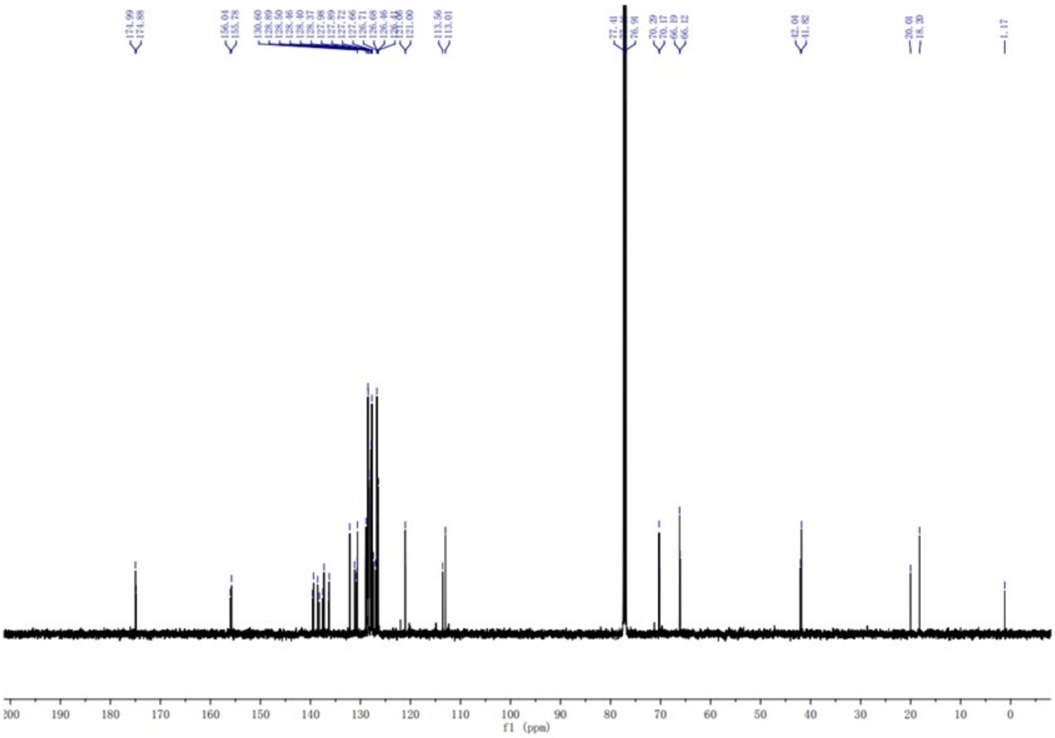

Figure S28. ^{13}C NMR (126 MHz, $CDCl_3$) spectrum of **8**.

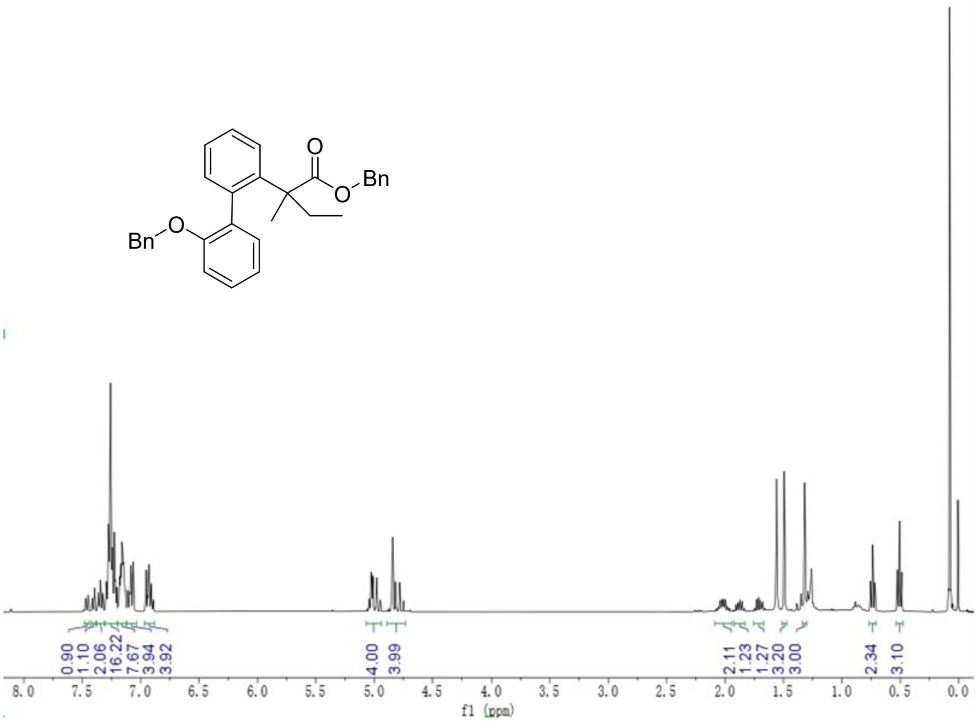

Figure S29. ^{1}H NMR (126 MHz, $CDCl_3$) spectrum of **9**.

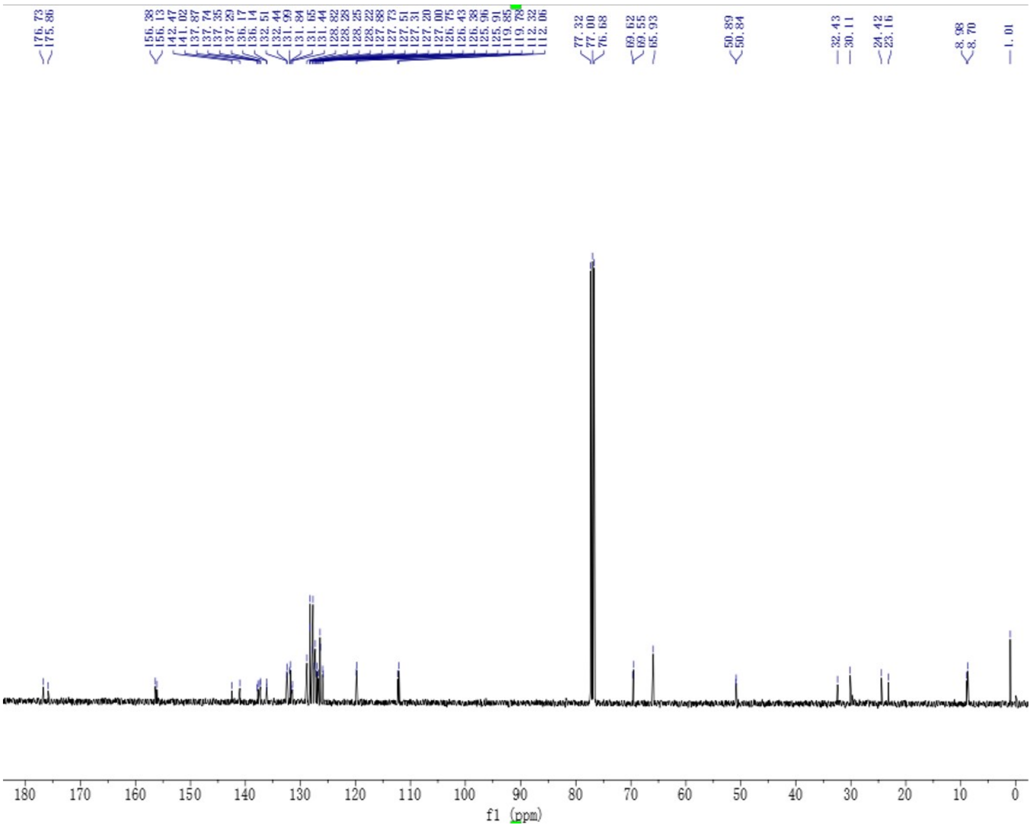

Figure S30. ^{13}C NMR (101 MHz, $CDCl_3$) spectrum of **9**.

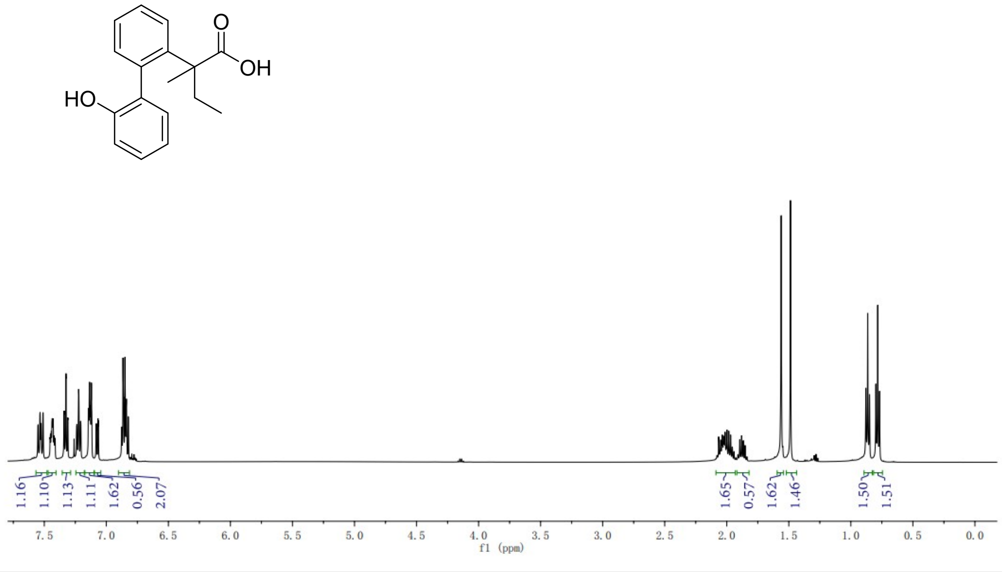

Figure S31. ^{1}H NMR (500 MHz, $CDCl_3$) spectrum of **10**.

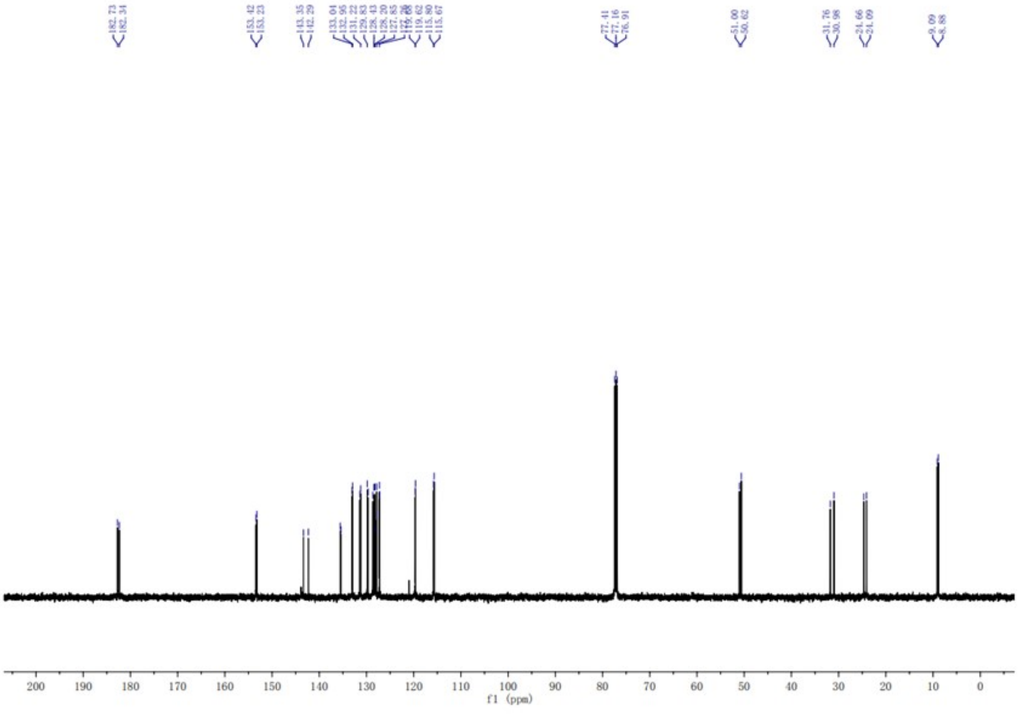

Figure S32. ^{13}C NMR (126 MHz, $CDCl_3$) spectrum of **10**.

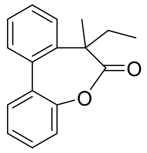

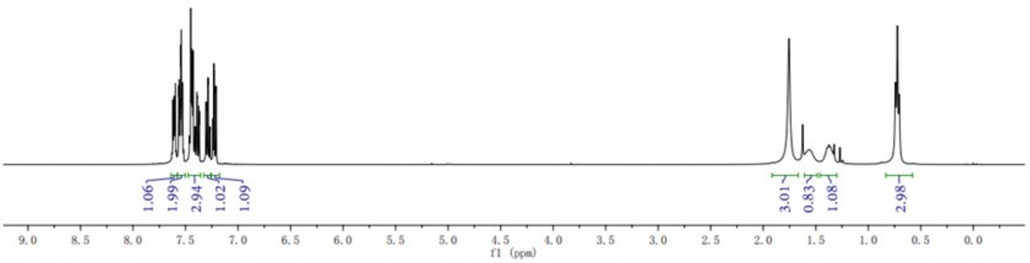

Figure S33. ^{1}H NMR (400 MHz, $CDCl_3$) spectrum of **5f**.

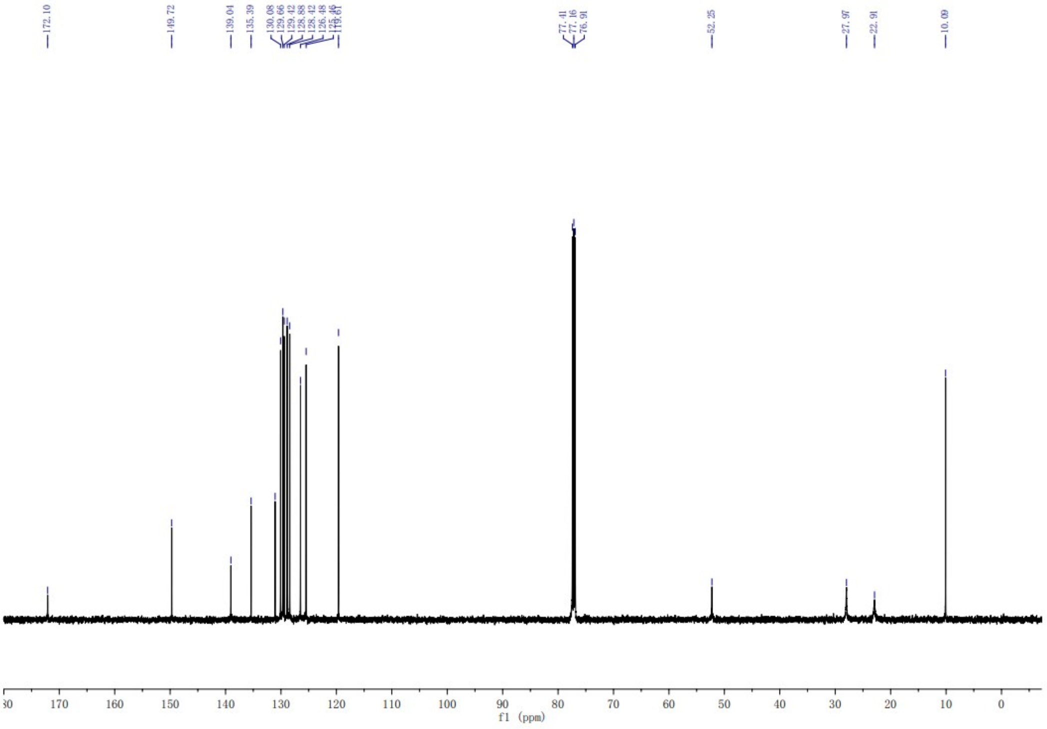

Figure S34. ^{13}C NMR (101 MHz, $CDCl_3$) spectrum of **5f**.

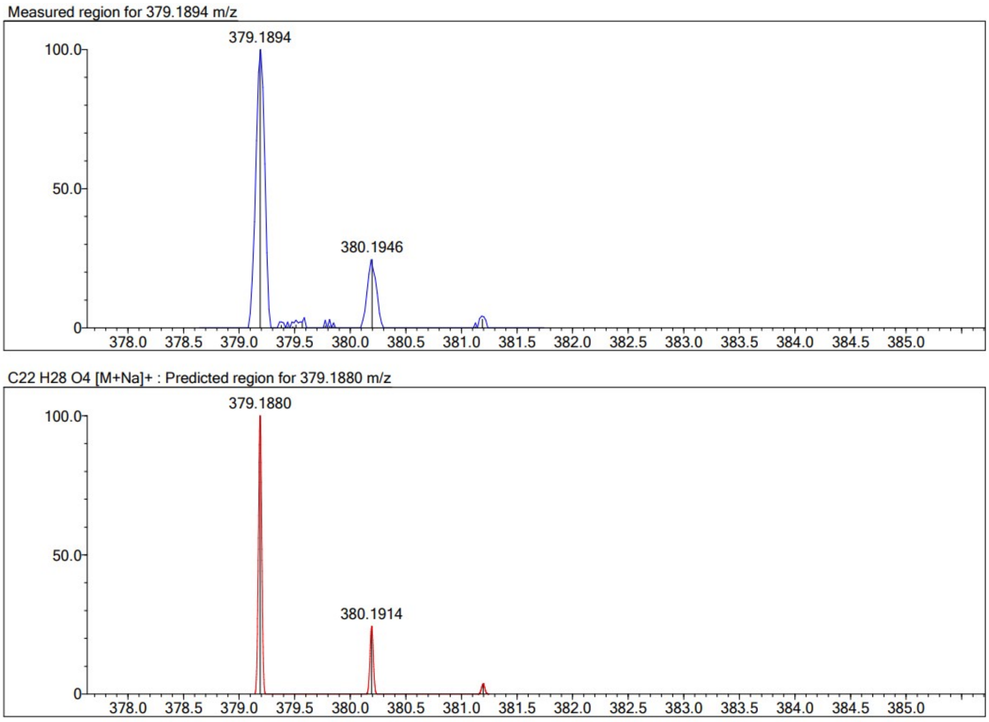

Figure S35. HRMS spectrum of compound **4b**.

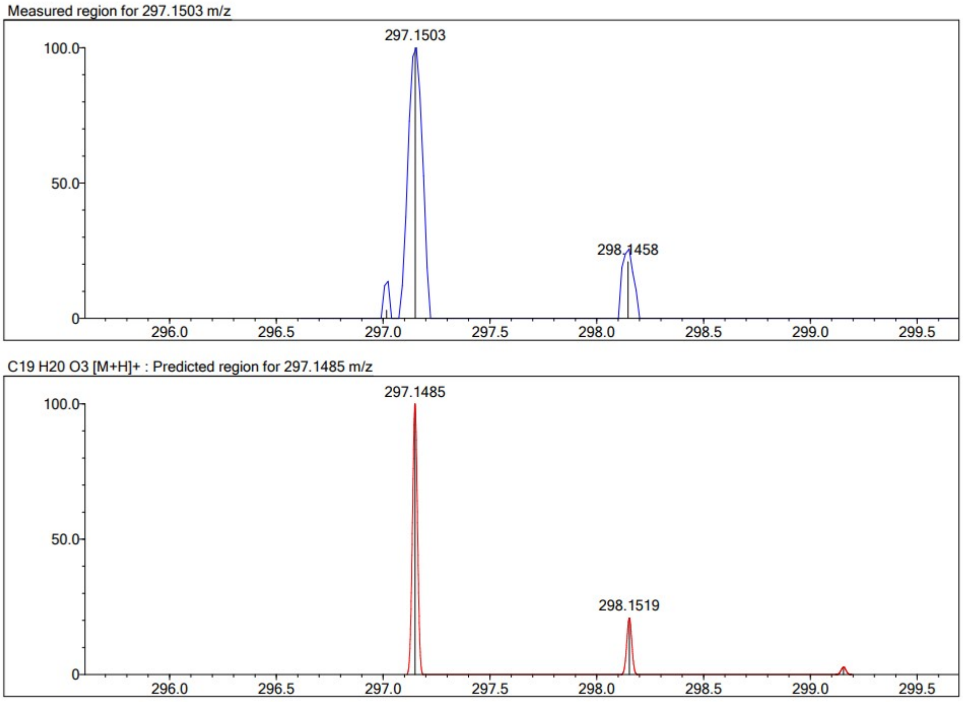

Figure S36. HRMS spectrum of compound **5b**.

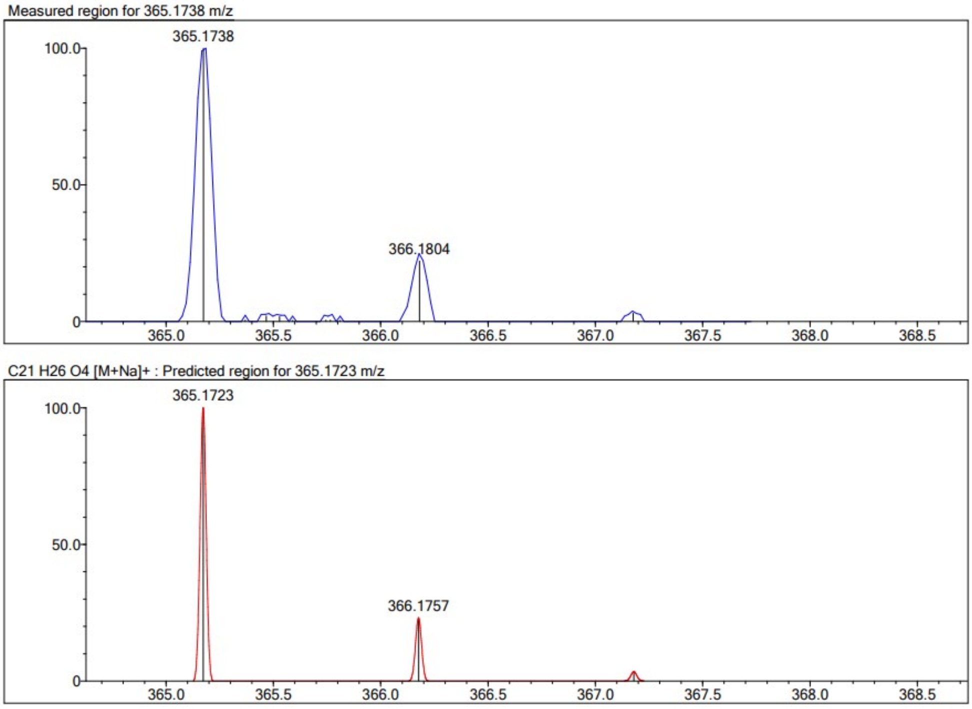

Figure S37. HRMS spectrum of compound **4c**.

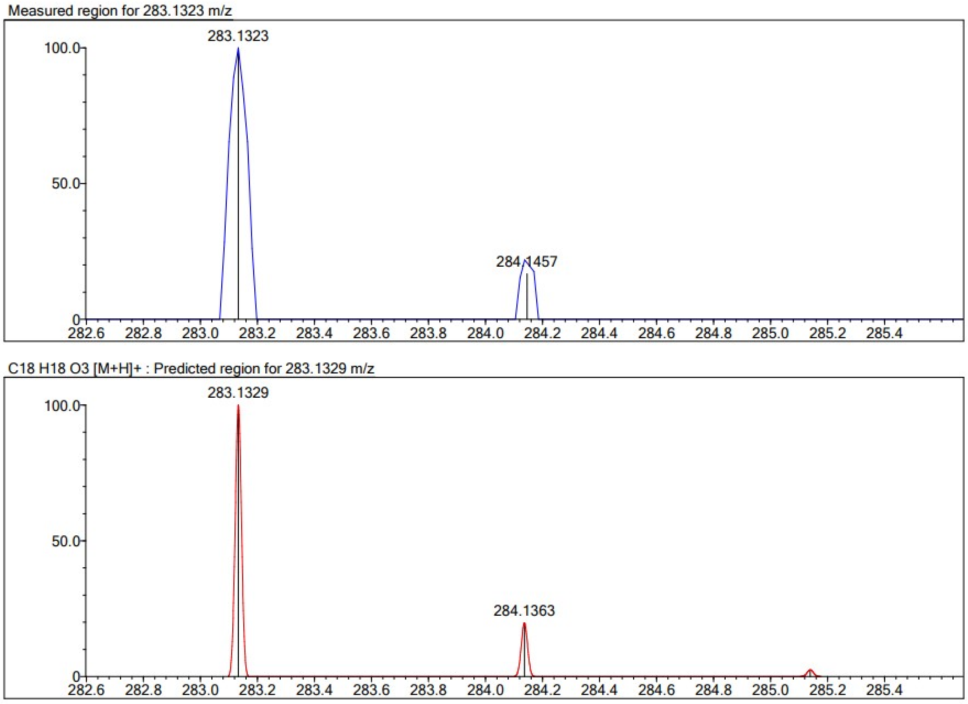

Figure S38. HRMS spectrum of compound **5c**.

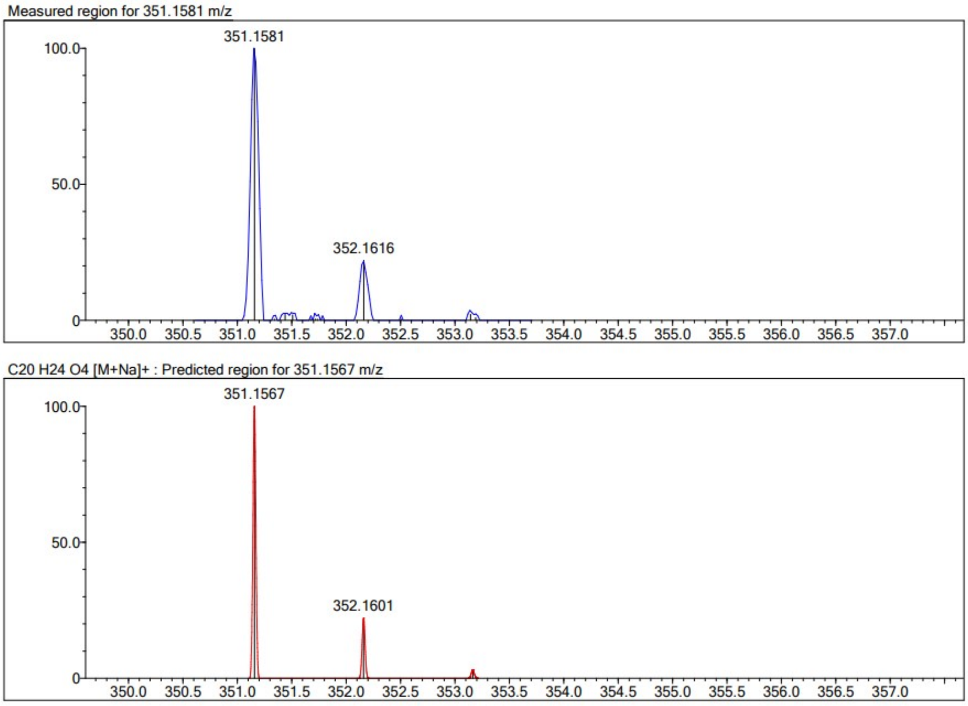

Figure S39. HRMS spectrum of compound **4d**.

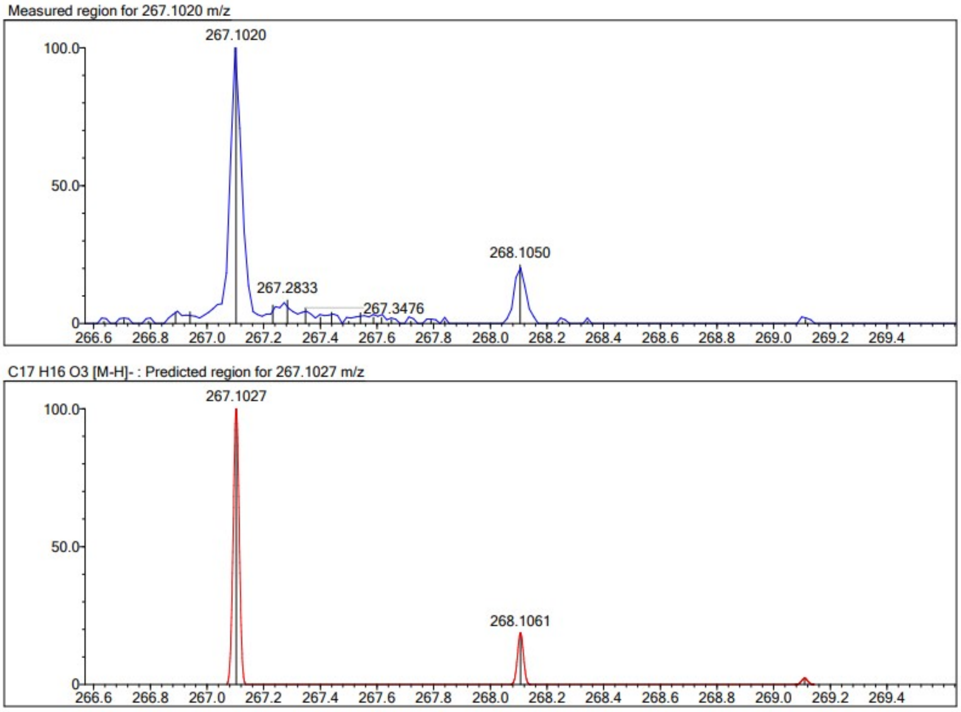

Figure S40. HRMS spectrum of compound **5d**.

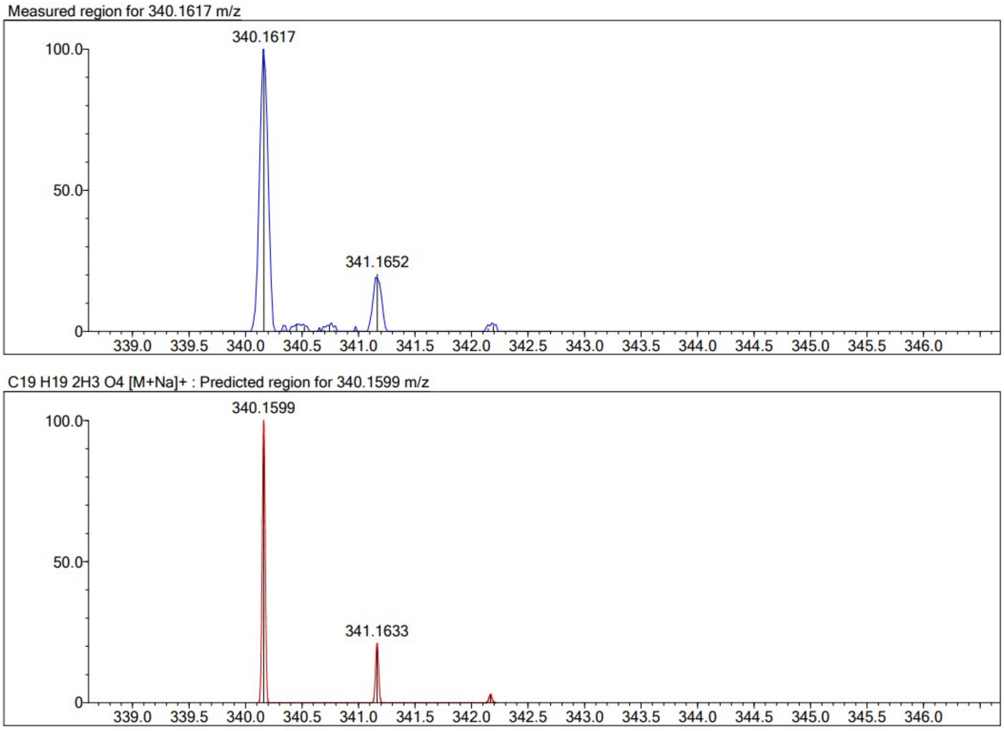

Figure S41. HRMS spectrum of compound **4e**.

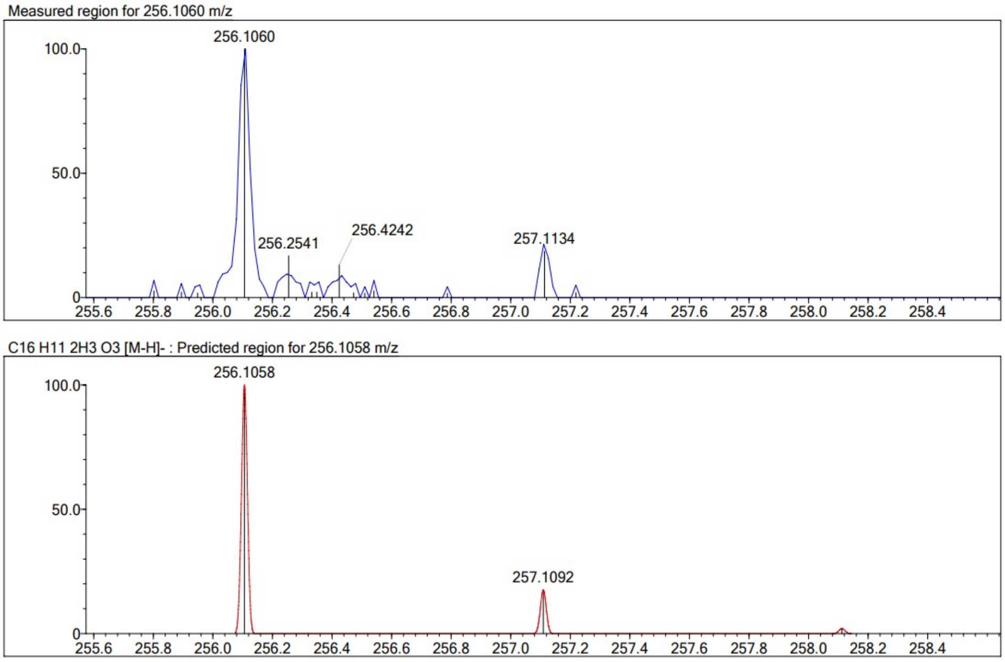

Figure S42. HRMS spectrum of compound **5e**.

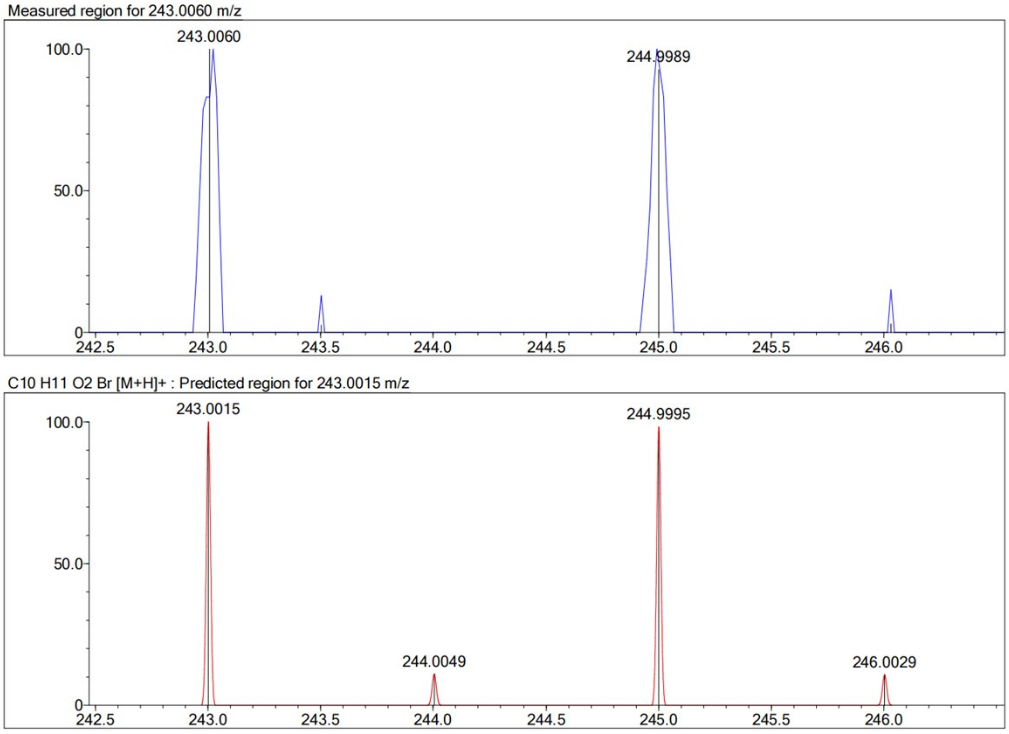

Figure S43. HRMS spectrum of compound **3**.

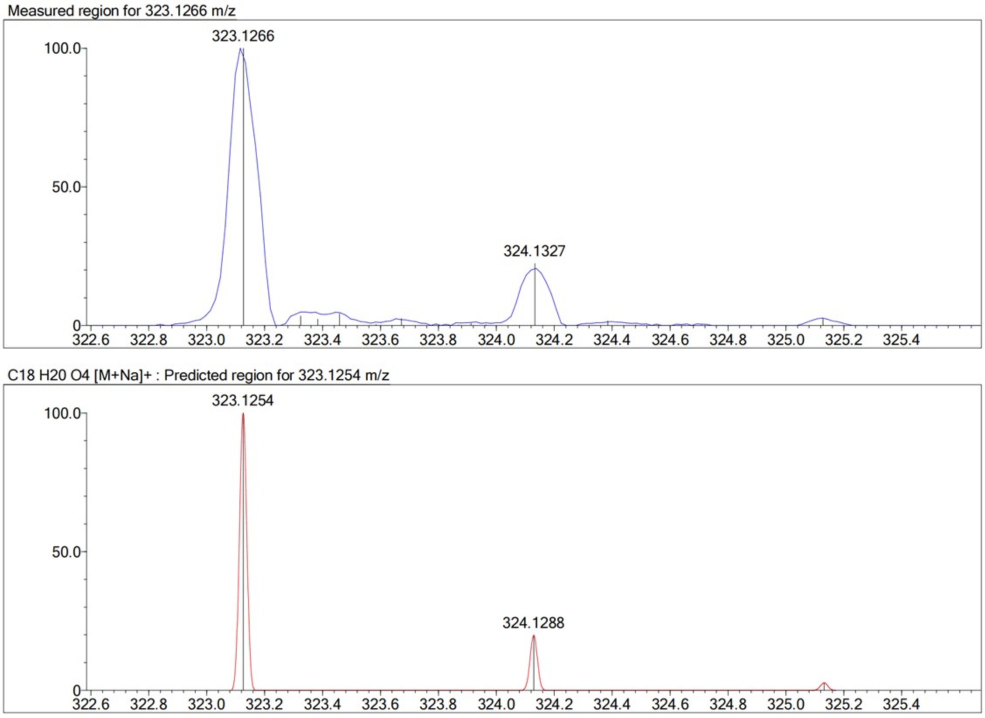

Figure S44. HRMS spectrum of compound **4a**.

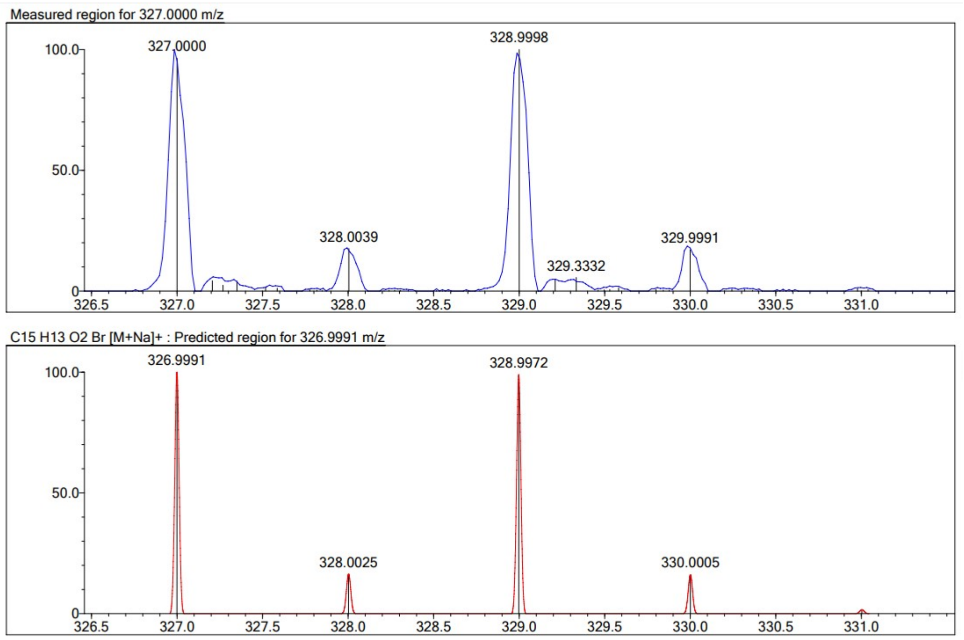

Rank	Score	Formula (M)	Ion	Meas. m/z	Pred. m/z	Df. (mDa)	Df. (ppm)	Iso	DBE
1	62.17	C15 H13 O2 Br	[M+Na]+	327.0000	326.9991	0.9	2.75	65.01	9.0

Figure S45. HRMS spectrum of compound **6**.

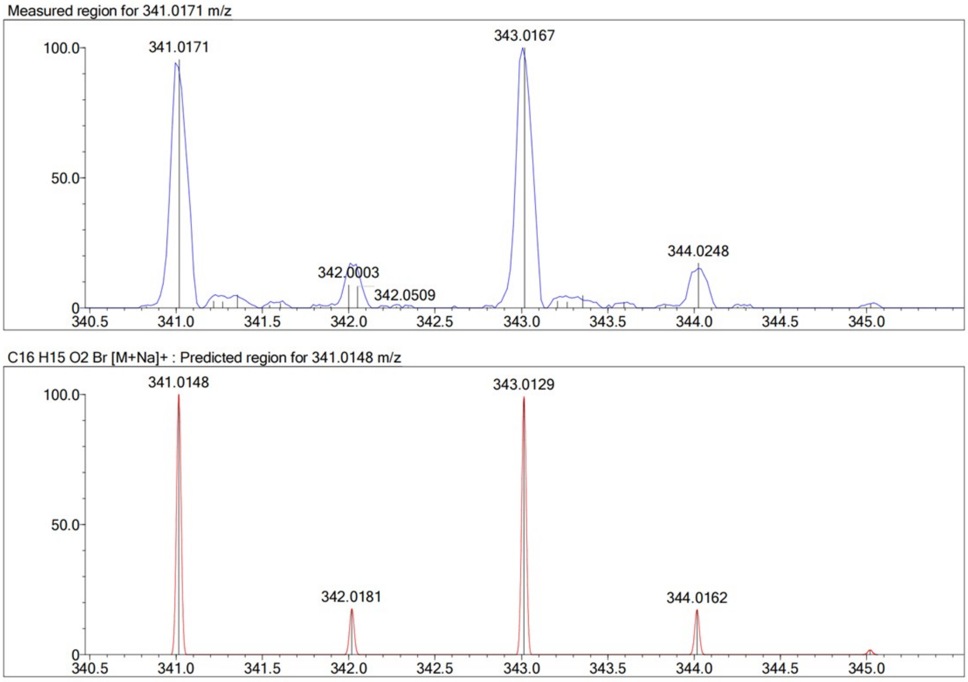

Figure S46. HRMS spectrum of compound **7**.

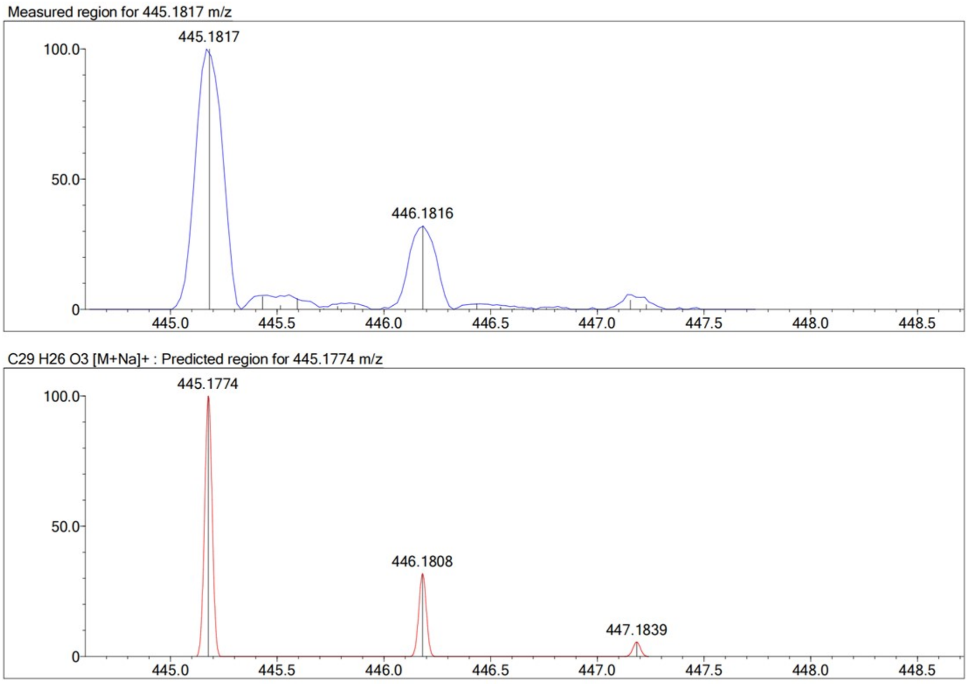

Figure S47. HRMS spectrum of compound **8**.

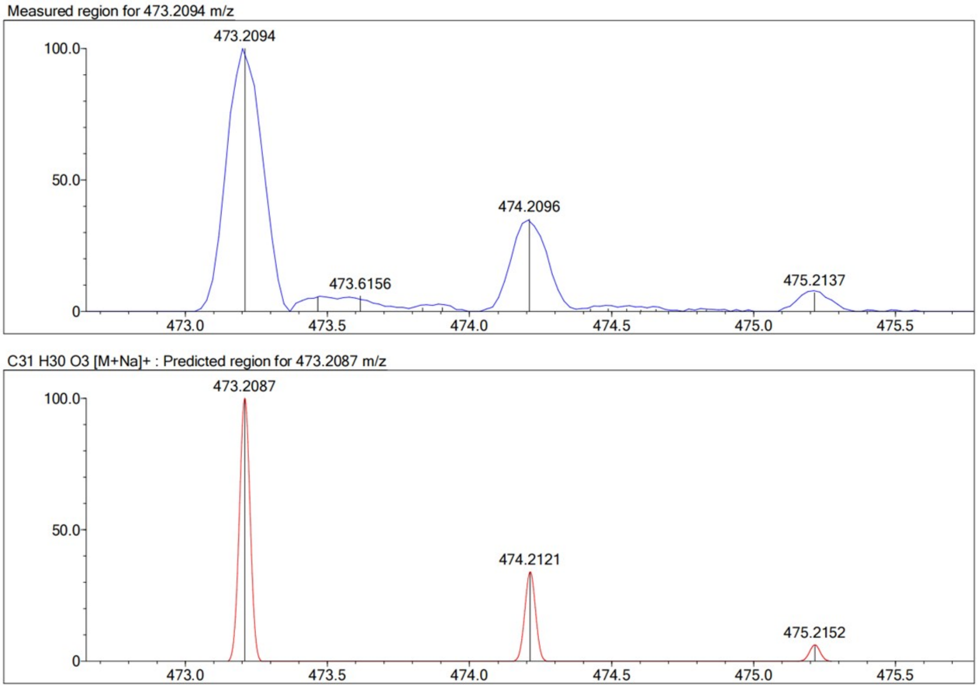

Figure S48. HRMS spectrum of compound **9**.

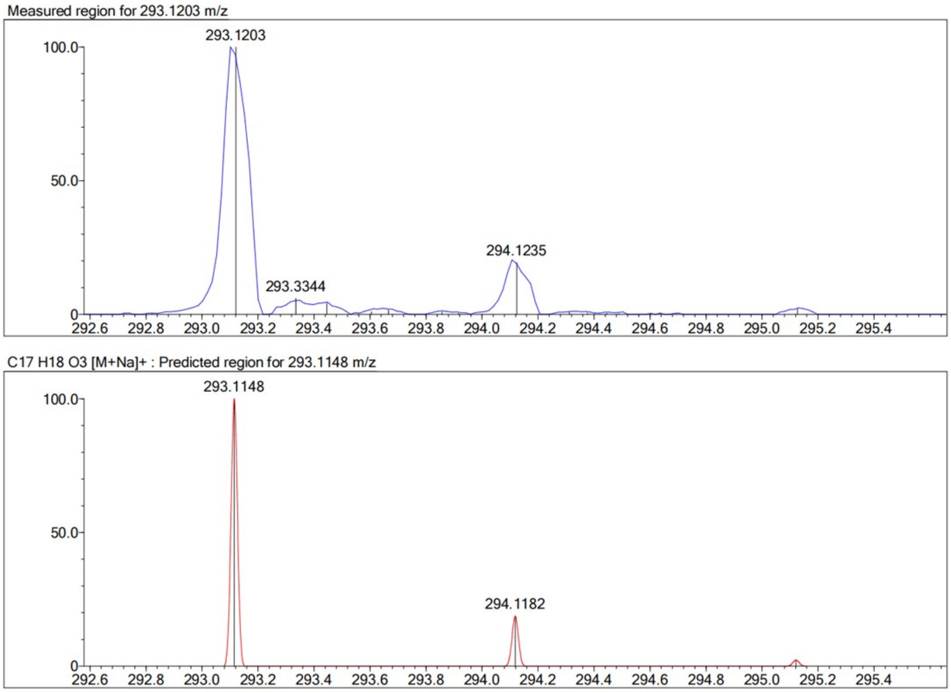

Figure S49. HRMS spectrum of compound **10**.

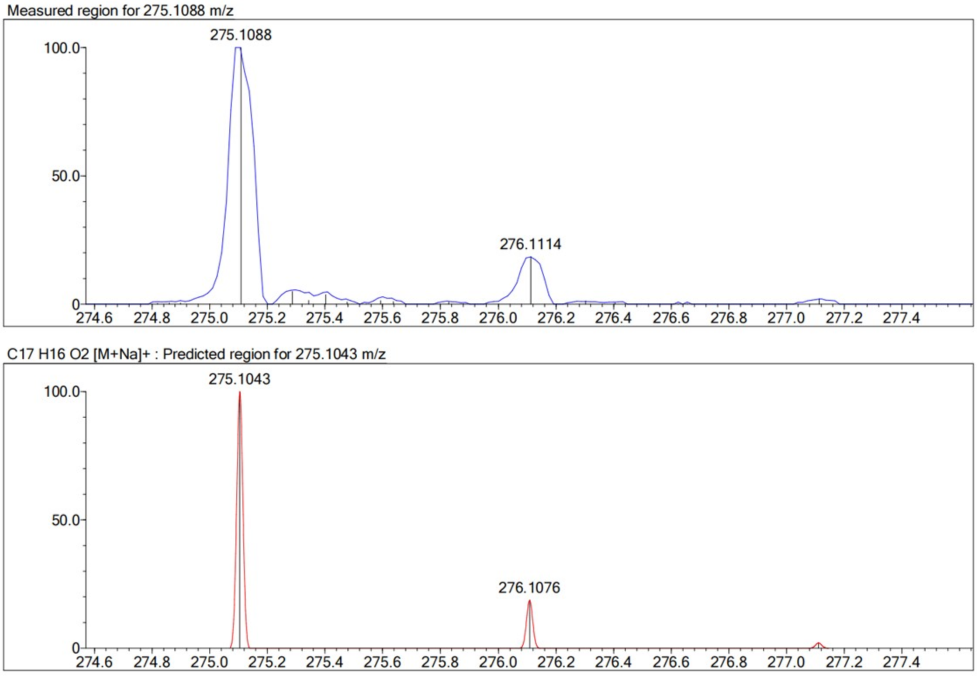

Figure S50. HRMS spectrum of compound **5f**.

IRI of 5d

The interaction region indicator (IRI) was achieved with the help of the Multiwfn program.[5,6] The isosurface map of IRI=1.0 of both major and minor conformations of **5d** are shown in Fig. S51 and Fig. S52. The chemical bonds, weak interactions and the intensity can be seen through the color of the IRI isosurfaces. The standard coloring method and chemical explanation are shown in Fig. S53. As shown in the Fig. S51 and Fig. S52, two configurations of **5d** both have similar weak interactions between the hydroxy group and the hydrogen of the benzene ring. Furthermore, the groups on the equatorial bonds of the quaternary carbons have weak interactions and steric effect with nearby atoms. It is worth noting that the ethyl group on the axial bond (Fig. S51) produces larger areas of weak interactions with the benzene ring than the methyl group (Fig. S52), which is indicated by the larger area of isosurface marked in green. It may lead to the lower energy of the conformation where the ethyl group is on the axial position.

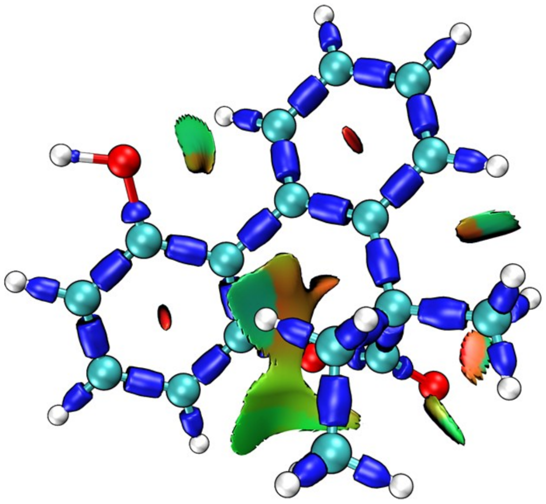

Figure S51. Isosurface map of IRI of the major conformation of **5d**.

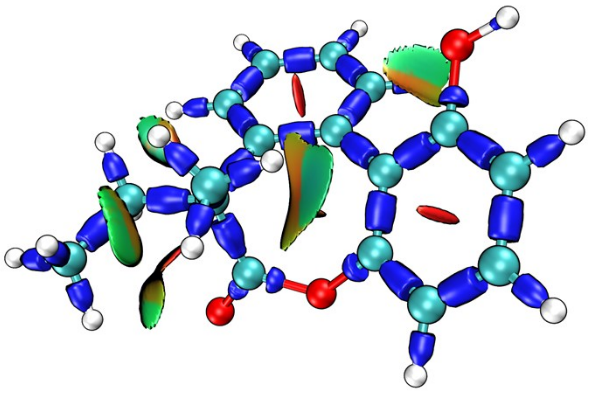

Figure S52. Isosurface map of IRI of the minor conformation of **5d**.

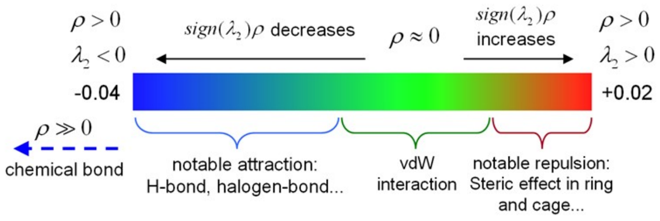

Figure S53. The standard color bar and chemical explanation of the IRI.

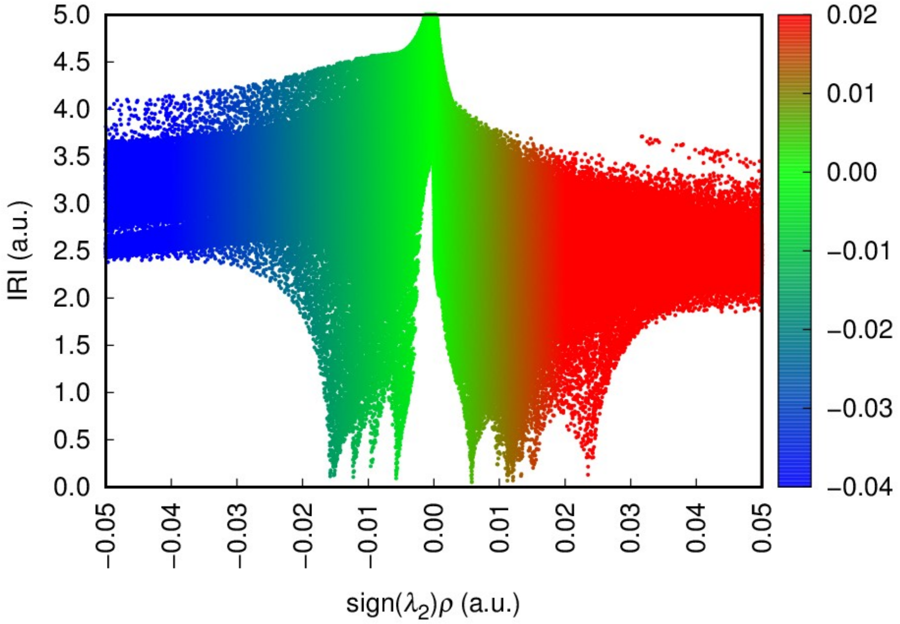

Figure 54. Scatter map of the major conformer of **5d** between IRI and sign(λ_2)ρ.

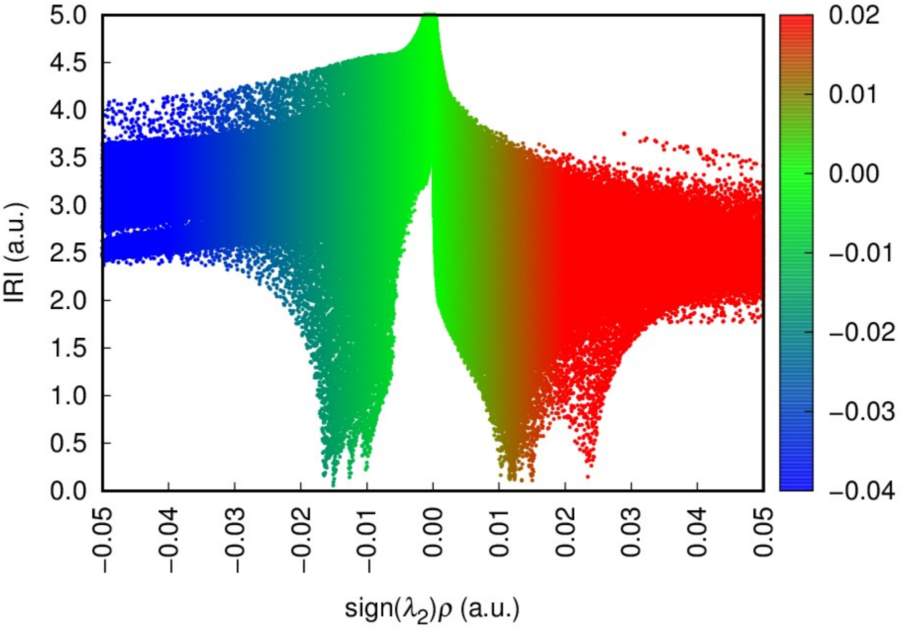

Figure 55. Scatter map of the minor conformer of **5d** between IRI and sign(λ_2)ρ.

Reference

1. P. Müller, R. Herbst-Irmer, A. L. Spek, T. R. Schneider, M. R. Swaya, Crystal Structure Refinement: A Crystallographer's Guide to SHELXL. Oxford University Press Inc. New York, 2006.
2. M. J. Frisch, G. W. Trucks, H. B. Schlegel, G. E. Scuseria, M. A. Robb, J. R. Cheeseman, G. Scalmani, V. Barone, G. A. Petersson, H. Nakatsuji, X. Li, M. Caricato, A. V. Marenich, J. Bloino, B. G. Janesko, R. Gomperts, B. Mennucci, H. P. Hratchian, J. V. Ortiz, A. F. Izmaylov, J. L. Sonnenberg, D. Williams-Young, F. Ding, F. Lipparini, F. Egidi, J. Goings, B. Peng, A. Petrone, T. Henderson, D. Ranasinghe, V. G. Zakrzewski, J. Gao, N. Rega, G. Zheng, W. Liang, M. Hada, M. Ehara, K. Toyota, R. Fukuda, J. Hasegawa, M. Ishida, T. Nakajima, Y. Honda, O. Kitao, H. Nakai, T. Vreven, K. Throssell, J. A. Montgomery, Jr., J. E. Peralta, F. Ogliaro, M. J. Bearpark, J. J. Heyd, E. N. Brothers, K. N. Kudin, V. N. Staroverov, T. A. Keith, R. Kobayashi, J. Normand, K. Raghavachari, A. P. Rendell, J. C. Burant, S. S. Iyengar, J. Tomasi, M. Cossi, J. M. Millam, M. Klene, C. Adamo, R. Cammi, J. W. Ochterski, R. L. Martin, K. Morokuma, O. Farkas, J. B. Foresman, and D. J. Fox, Gaussian, Inc., Wallingford CT, 2016. Gaussian 16, Revision A.03.
3. K. Fukui, The path of chemical reactions - The IRC approach. *Acc. Chem. Res.*, 1981,**14**, 363.
4. Y. Zhang, Z. Chang, H. Zhao, S. Crespi, B. L. Feringa, D. Zhao, *Chem*, 2020, **6**, 2420.
5. Tian Lu, Feiwu Chen, Multiwfn: A Multifunctional Wavefunction Analyzer, *J. Comput. Chem.*, 2012, **33**, 580-592.
6. Tian Lu, Qinxue Chen, Interaction Region Indicator (IRI): A Simple Real Space Function Clearly Revealing Both Chemical Bonds and Weak Interactions, *Chemistry—Methods*, 2021, **1**, 231-239.
